# Disruption of primary ciliary prostaglandin E_2_ signaling by transforming growth factor-β1 impairs endometrial receptivity

**DOI:** 10.1186/s12929-026-01233-2

**Published:** 2026-03-11

**Authors:** Huan-Tzu Hou, Wan-Ning Li, Ting-Chien Lin, Chih-Wei Lin, Po-Hung Pan, Chih-Jhen Lee, Yi-Chen Chen, Po-Fan Chen, Chia-Yih Wang, Meng-Hsing Wu, Shaw-Jenq Tsai

**Affiliations:** 1 Institute of Basic Medical Sciences, College of Medicine, National Cheng Kung University, Tainan, Taiwan; 2 Department of Obstetrics and Gynecology, National Cheng Kung University Hospital, Tainan, Taiwan; 3 Department of Physiology, College of Medicine, National Cheng Kung University, 1 University Road, Tainan, 70101 Taiwan; 4 Department of Anatomy and Cell Biology, College of Medicine, National Cheng Kung University, Tainan, Taiwan; 5 Department of Biomedical Sciences, College of Science, National Chung Cheng University, 168, Sec. 1, University Road, Chiayi, 621301 Taiwan

**Keywords:** Decidualization, Endometriosis, Infertility, PGE_2_, Primary cilia, TGF-β1

## Abstract

**Background:**

Infertility affects one in six individuals worldwide despite the advancement of assisted reproductive technologies. Successful embryo implantation is the first step of pregnancy, which relies on the establishment of a receptive uterine microenvironment. However, the mechanisms governing uterine receptivity and implantation failure remain incompletely characterized. Primary cilia serve as key cellular signaling hubs, yet their contribution to human decidualization and uterine receptivity remains largely unexplored.

**Methods:**

Primary cultured human endometrial stromal cells (ESCs) were used to investigate the mechanisms of decidualization, functions of primary cilia, and effects of transforming growth factor-β (TGF-β) in inhibiting prostaglandin E_2_ (PGE_2_)-induced decidualization. Human endometrial tissues (n = 108) were used to evaluate the clinicopathological parameters. The percentage of ciliated cells and cilia length were determined by immunofluorescent staining and AI-assisted quantification. Pseudopregnancy and pregnancy mouse models were employed to assess the effects of TGF-β1 on uterine receptivity and implantation outcomes.

**Results:**

Prostaglandin E_2_, through binding to the EP4 receptor located at the primary cilium, stimulates ESC decidualization, which is augmented by 17β-estradiol and progesterone. Loss of ciliogenesis by genetic or pharmacological inhibition impairs decidualization. Proinflammatory cytokines such as TGF-β inhibit ciliogenesis and thus markedly attenuate PGE_2_-mediated decidualization. Mechanistically, TGF-β1 suppressed chicken ovalbumin upstream promoter transcription factor II and its downstream effector kinesin family member 3B, thereby inhibiting ciliogenesis and PGE₂-EP4 signaling. In mice, intrauterine administration of TGF-β1 impaired implantation, while TGF-β receptor blockade restored ciliogenesis, decidualization, and fertility. In women with endometriosis, ESCs displayed shortened cilia and reduced decidual response, which are due to elevated uterine and peritoneal TGF-β1-mediated suppression of ciliogenesis. Finally, women who failed to conceive after in vitro fertilization-embryo transfer (IVF-ET) have shorter and fewer primary cilia in ESCs. Receiver operating characteristic curve analysis demonstrated that both cilia length (AUC = 0.86) and ciliation frequency (AUC = 0.79) can serve as biomarkers for endometrial receptivity, providing predictive value for reproductive outcomes independent of ovarian reserve.

**Conclusions:**

Endometrial primary cilia are indispensable for decidualization and are potential biomarkers for predicting endometrial receptivity. Targeting TGF-β signaling to restore ciliated cell number and ciliary length may serve as a potential therapeutic strategy to improve fertility outcomes.

**Supplementary Information:**

The online version contains supplementary material available at 10.1186/s12929-026-01233-2.

## Background

Infertility is a major threat to species sustainability. A recent World Health Organization report states that one in six people suffers from infertility [1], with even higher rates in developed countries. A receptive endometrium is essential for embryo implantation and pregnancy success, and its failure is a key contributor to infertility and early pregnancy loss, even in in vitro fertilization-embryo transfer (IVF-ET) cycles [2]. Decidualization, the differentiation of endometrial stromal cells (ESCs) into decidual cells, is a prerequisite for establishing uterine receptivity and occurs naturally during the mid-secretory phase of the menstrual cycle [2]. Signaling pathways involving cyclic adenosine monophosphate (cAMP) and protein kinase A (PKA) play important roles in promoting the expression of decidual genes such as insulin-like growth factor-binding protein 1 (IGFBP1), Forkhead box protein O1 (FOXO1), and Prolactin (PRL) [3, 4]. FOXO1 is a master transcription factor regulating genes involved in decidualization either alone or as a co-regulator [5]. IGFBP1 not only functions as a binding protein to regulate the availability of IGF-1 and IGF-II, but it also exerts IGF-independent function to induce prolactin expression via binding to α5β1 integrin [6]. However, the uterine factors regulating endometrial decidualization or causing implantation failure remain largely uncharacterized.

Primary cilia are solitary, microtubule-based organelles that function as signaling hubs on nearly all mammalian cells [7]. Multiple signaling pathways, including Hedgehog, Wnt, Notch, and cAMP, are localized within the primary cilium [7]. Impaired ciliogenesis can disrupt receptor localization and intracellular signal transduction, leading to abnormal cellular differentiation and function [8]. The functional relevance of primary cilia has been demonstrated in many physiological processes, including the development of the placenta. In trophoblasts, primary cilia coordinate endocrine-derived vascular endothelial growth factor signaling, promoting the expression of matrix metallopeptidases required for invasion and proper placental formation [9]. Disruption of trophoblast cilia impairs their migratory and invasive capacity, resulting in placental defects [10]. Furthermore, reduced ciliation or shortened cilia in mesenchymal stromal cells of the chorionic villi have been linked to pregnancy complications such as preeclampsia and recurrent miscarriage [11, 12]. Although these findings emphasize the importance of primary cilia in placental development and maintenance of pregnancy, their specific contribution to human uterine receptivity and the decidualization of endometrial stromal cells remains incompletely understood.

Endometriosis is a gynecological disorder frequently associated with infertility and affects approximately 25 to 50 percent of women with subfertility [13, 14]. Several factors contribute to reduced fertility in women with endometriosis, including poor oocyte quality, a pro-inflammatory uterine environment, and impaired endometrial function. Studies have shown downregulation of key decidual markers, such as FOXO1 and Homeobox A10 (HOXA10), in the endometrium of women with endometriosis [15, 16]. ESCs derived from endometriosis patients also exhibit reduced decidualization capacity in vitro [17]. However, the underlying mechanisms that impair decidualization and uterine receptivity in endometriosis remain largely undefined.

In this study, we investigate the functional role of primary cilia in the decidualization of human ESCs and explore how defective ciliogenesis may contribute to implantation failure and infertility. Inflammatory cytokines, including transforming growth factor-β1 (TGF-β1), are elevated in the peritoneal and uterine environments of women with endometriosis and have been implicated in impairing endometrial function [18, 19]. We therefore examined whether TGF-β1 disrupts cilia-dependent signaling pathways essential for decidualization and endometrial receptivity.

## Methods

### Clinical samples from patients

Endometrial tissues, uterine fluid, and peritoneal fluid were obtained from women with or without endometriosis undergoing hysteroscopic or laparoscopic surgery at National Cheng Kung University Hospital. Control patients included those with benign conditions (e.g., endometrial or cervical polyps, leiomyomas, or tubal obstruction) but without endometriosis. Women with adenomyosis were excluded. For IVF-ET samples, endometrial biopsies were collected before embryo transfer cycles. Informed consent was obtained from all participants, and the study was approved by the Clinical Research Ethics Committee at National Cheng Kung University Medical Center. Detailed collection protocols. Patient information is summarized in Tables S1–3. More detailed information is described in the Supplementary Information, Methods.

### Primary ESC isolation and treatment

Human ESCs were isolated as previously described [20]. Tissues were digested with 2 mg/ml collagenase IV at 37 °C for 1 h, filtered (70-μm and 40-μm), and stromal cells were enriched by brief adherence. Cells were cultured in DMEM/F12 supplemented with 10% FBS and 1% (1 ×) penicillin–streptomycin at 37 °C, 5% CO₂. For the induction of ciliogenesis and cytokine treatment, ESCs were cultured in a serum-free medium for 48 h in the presence or absence of recombinant TGF-β1 (5 or 10 ng/ml, PeproTech, 100-21). For the in vitro decidualization, ESCs were treated with DMEM/F12 containing 10 nM 17β-estradiol (Sigma-Aldrich, E2758), 1 μM medroxyprogesterone acetate (Sigma-Aldrich, M1629), and 50 μM 8-bromo-cAMP (Sigma-Aldrich, B5368), the EPC medium, under serum-free conditions or with 10% charcoal-stripped fetal bovine serum to induce decidualization for 4–6 days. In the PGE_2_-induced decidualization system, ESCs were treated with EPG medium, which is a mixture of 10 nM 17β-estradiol, 1 μM medroxyprogesterone acetate, and 1 μM PGE_2_ (Cayman Chemical, 14010) in DMEM/F12 to induce decidualization for 24 h or 4 days. Detailed treatment protocols are provided in the Supplementary Information, Methods.

### RNA isolation, reverse transcription quantitative PCR (RT-qPCR), and Western blot analysis

Total RNA and proteins were extracted from ESCs lysed in TRIzol (Invitrogen, 15,596,018) reagent following the manufacturer’s protocol. The concentration of RNA was determined by measuring absorbance at 260 nm using a NanoDrop spectrophotometer (NanoDrop, ND-1000). Total RNA (500 ng) was reverse transcribed by incubating at 42 °C for 90 min, followed by 95 °C for 10 min. Two microliters of the resulting cDNA were utilized in SYBR Green (Applied Biosystems, 4,309,155)-based PCR amplification using the Applied Biosystems StepOnePlus™ Real-Time PCR System (Foster City, CA, USA). The sequences of primers used in this study are listed in Table S4. For Western blotting, protein concentration was assessed via the Lowry assay. Proteins (20 μg/sample) were then denatured at 95 °C with 6X sample buffer dye for 10 min, separated by electrophoresis on a polyacrylamide gel, and transferred onto polyvinylidene fluoride membranes. Following blocking with 5% non-fat milk in phosphate-buffered saline (PBS) with 0.05% Tween-20 (PBST) at room temperature for 1 h, the membrane was incubated with primary antibodies at 4 °C overnight. After washing with PBST three times, the membrane was incubated with a peroxidase-conjugated secondary antibody for 1 h at room temperature. Subsequently, the membrane was washed with PBST three times, and immunodetection was performed using an enhanced chemiluminescent substrate (PerkinElmer, NEL104001EA). The antibody information was listed in Table S5.

### Immunohistochemistry (IHC) staining and immunofluorescence staining

For IHC staining, paraffin-embedded samples (4 μm thick) were dewaxed and subjected to antigen retrieval in citrate buffer (pH 6.0). After blocking with Protein Block (Novocastra, RE7102) for 1 h at room temperature, samples were incubated overnight at 4 °C with primary antibodies. The next day, sections were washed with PBST and PBS, followed by incubation with Polymer or Post Primary block (Novolink, RE7260-K) for 1 h at room temperature. Signal was developed using AEC substrate buffer (Bio SB, BSB0014) and counterstained with hematoxylin (Leica, 351,521). For immunofluorescence staining, paraffin-embedded human endometrium or mouse uterus was processed as above for dewaxing and antigen retrieval. Rabbit anti-human ADP-ribosylation factor-like protein 13B (ARL13B) and mouse anti-human acetylated tubulin antibodies were used to stain primary cilia. For co-staining experiments, one of these markers was selected to pair with a third antibody based on host species compatibility to prevent cross-reactivity. After primary antibody incubation, sections were incubated with goat anti-rabbit IgG Alexa Fluor 488 (Invitrogen, A32731) and/or goat anti-mouse IgG Alexa Fluor 594 (Invitrogen, A32740) for 1 h, followed by Hoechst 33,258 nuclear staining (Invitrogen, H3569). Samples were washed with PBST and PBS, then mounted using Fluoromount-G (Invitrogen, 00-4958-02).

### Immunocytochemistry staining

For immunocytochemistry, cells grown on glass coverslips were fixed with 4% formaldehyde or methanol for 10 min at room temperature, then permeabilized with 0.3% Triton X-100 in PBS for 10 min. After blocking with 5% goat serum in 0.5% Tween-20/PBS for 1 h, cells were incubated overnight at 4 °C with primary antibodies diluted in 5% goat serum/PBS. Following three PBS washes, cells were incubated for 1 h at room temperature with goat anti-rabbit IgG Alexa Fluor 488 (Invitrogen, A32731) and/or goat anti-mouse IgG Alexa Fluor 594 (Invitrogen, A32740), together with Hoechst 33,258 nuclear stain (Invitrogen, H3569). Coverslips were washed and mounted using Fluoromount-G (Invitrogen, 00-4958-02).

### Measurement and quantification of primary cilia

The percentage of ciliated cells and cilia length in endometrial specimens and ESCs was measured using CiliaQ, a plugin for the Fiji platform. The detection and counting of nuclei in immunofluorescence images of endometrial specimens were performed with StarDist, another Fiji plugin. Primary cilia quantification in endometrial specimens involved analyzing 800-1,200 cells per field, including cilia longer than 0.46 µm. Cilia with lengths shorter than 0.46 µm are defined as ciliary bases, which were excluded from the quantification of cilia length and percentage of ciliated cells. Due to subject variations, the basal levels of percentages of ciliated cells range from 55 to 85% (69.9% ± 14.9%). In cultured ESCs, ten immunofluorescence images from each treatment group within a biological replicate were analyzed. The number of biological replicates was indicated in each quantified result.

### Enzyme-linked immunosorbent assay (ELISA)

For TGF-β1 measurement, the levels of TGF-β1 in patients’ peritoneal fluid or uterine fluid were assessed using LEGEND MAXTM Total TGF-β1 ELISA Kit (BioLegend, 436,707) according to the manufacturer’s instructions. The peritoneal fluid or uterine fluid specimen was diluted with a dilution buffer provided by the kit. For PRL analysis, the concentration of PRL in cultured medium was assessed by using the human Prolactin (human) ELISA Kit (Cayman Chemical, 500,730).

### siRNA transfection

For siRNA transfection, 2 × 10^5 cells were seeded per well in 6-well plates. siRNAs targeting chicken ovalbumin upstream promoter transcription factor II (COUP-TFII) (Thermo Fisher, s41021 and s41023) or Kinesin family member 3B (KIF3B) (Thermo Fisher, s17928 and s17929) were diluted in Opti-MEM™ (Gibco), and Lipofectamine™ 2000 (Invitrogen) was diluted separately. After 5 min, Lipofectamine was mixed with siRNAs and incubated for 15 min to form complexes, which were added before the cells attached. After 16–18 h, the medium was replaced with serum-free DMEM/F12. Knockdown efficiency was verified by Western blotting.

### Pseudopregnancy and pregnancy mouse models

Eight-week-old female C57BL/6 mice were used. For pseudopregnancy, females were mated with vasectomized males; the day of vaginal plug was 0.5 dpc. At 0.5 dpc, under isoflurane, intrauterine injections of TGF-β1 (10 ng in 25 μl PBS per horn) or PBS were performed via small dorsal incisions; uteri were collected at 4.5 dpc. For pregnancy, females were mated with fertile males; TGF-β1 or PBS was injected at 1.5 dpc using the same procedure, and implantation was assessed at 7.5 dpc after intravenous 1% Evans Blue. To inhibit TGF-β signaling, SB431542 (4 mg/ml, 25 μl per horn) or 20% DMSO vehicle was administered 30 min before TGF-β1. All procedures were approved by the Institutional Animal Care and Use Committee of the College of Medicine, National Cheng Kung University (Approval No: IACUC-111145). Detailed treatment protocols are provided in the Supplementary Information, Methods.

### Statistical analysis

Data were presented as mean ± standard deviation (SD), and two-sided statistical analyses were performed using GraphPad Prism 7.0 (GraphPad Software). Data normality was assessed using the Shapiro–Wilk test. For comparisons between two groups, Student’s *t*-test was used for normally distributed data, and the Mann–Whitney *U* test was used for non-normally distributed data. For comparisons involving multiple groups with a single factor, one-way ANOVA followed by Tukey’s post-hoc test was used. For experiments involving more than two groups with multiple factors, two-way ANOVA was applied to assess main effects and interactions, followed by Tukey’s post-hoc test for multiple comparisons. Pearson’s correlation was conducted to evaluate the relationships between peritoneal TGF-β1, average cilia length, and percentage of ciliated cells, as well as between the expression of p-CREB and both average cilia length and the percentage of ciliated cells. The Chi-square test was employed to analyze the implantation rate in mice. A P value of < 0.05 was considered as statistical significance.

## Results

### Primary ciliary EP4 mediates the decidualizing effect of PGE_2_

**Fig. 1:**
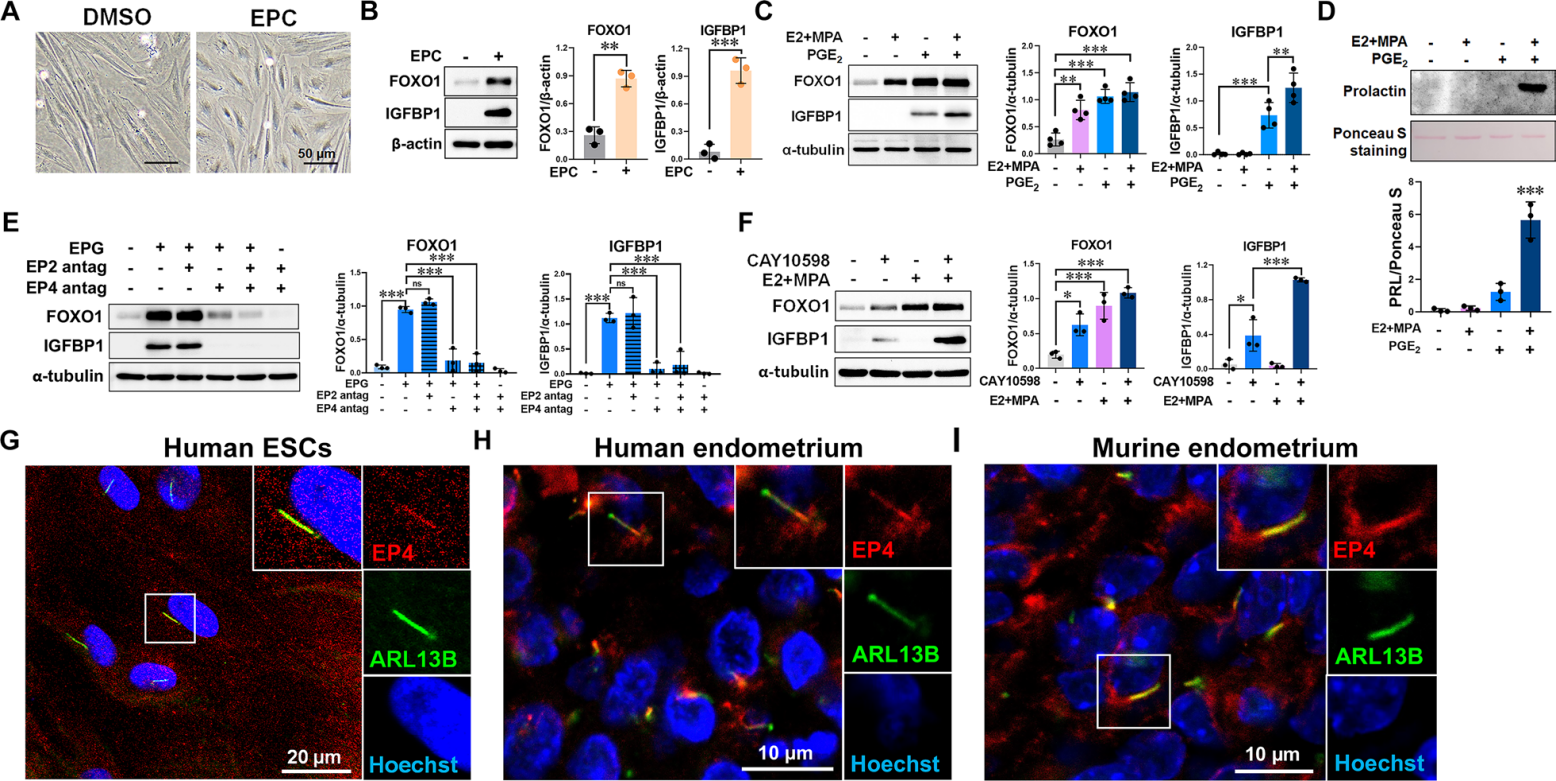
PGE_2_ stimulates ESC decidualization. **A** Bright-field of ESCs treated with EPC or DMSO for 6 days. Scale bar = 50 μm. **B** Western blot images and quantification of FOXO1 and IGFBP1 expression in ESCs with or without EPC treatment for 6 days (n = 3 biological replicates). **C** Western blot images and quantification of FOXO1 and IGFBP1 expression in ESCs after treatment with PGE_2_ (1 μM) and/or medroxyprogesterone acetate (MPA, 1 μM) combined with 17β-estradiol (E2, 10 nM) for 4 days (n = 4 biological replicates). **D** Western blot images and quantification of prolactin expression in the media collected from ESCs after treatment with PGE_2_ (1 μM) and/or MPA (1 μM) combined with 1E2 (10 nM) for 4 days (n = 3 biological replicates). **E** Western blot images and quantification of FOXO1 and IGFBP1 in ESCs pretreatment with an EP2 antagonist (EP2 antag, 10 μM) and/or an EP4 antagonist (EP4 antag, 10 μM), followed by treatment with E2 (10 nM), MPA (1 μM), and PGE_2_ (1 μM) for 4 days (n = 3 biological replicates). **F** Western blot images and quantification of FOXO1 and IGFBP1 in ESCs after treatment with CAY10598 (1 μM), with or without E2 (10 nM) and MPA (1 μM) for 4 days (n = 3 biological replicates).** G–I** Confocal images showing the localization of EP4 (red) and ARL13B (green) in human ESCs (**G**), human endometrium (secretory phase, **H**), and murine endometrium (4.5 days post-coitum, **I**). Nuclei were stained with Hoechst (blue). *p ≤ 0.05, **p ≤ 0.01, ***p ≤ 0.001 by Student’s *t*-test, one-way ANOVA with Tukey’s multiple test, or two-way ANOVA with Tukey’s multiple test (panels C and F)

To investigate the molecular mechanism of decidualization, ESCs isolated from human endometria were stimulated with EPC medium containing 17β-estradiol (E2), medroxyprogesterone acetate (MPA), and 8-bromo-cAMP. This treatment induced characteristic morphological changes and upregulated the decidual markers IGFBP1 and FOXO1 (Fig. 1A, B). Given that prostaglandin E₂ (PGE_2_) is a known regulator of ESC differentiation and a stimulator of intracellular cAMP production through E-type prostanoid (EP) receptors [21], we examined its effect on decidualization. PGE₂ treatment significantly upregulated the decidualization markers IGFBP1, FOXO1, and prolactin (Fig. 1C, D; Fig. S1A). Notably, while E_2_ + MPA induced FOXO1 expression, they failed to stimulate IGFBP1 at either the mRNA or protein level (Fig. 1C; Fig. S1A). Instead, E_2_ + MPA exerted a permissive effect, sensitizing the cells to markedly augment PGE₂-mediated IGFBP1 upregulation (Fig. 1C; Fig. S1A). This hormone cocktail (E2 + MPA + PGE_2_), hereafter referred to as EPG, effectively induced decidualization (Fig. S1B). Because PGE₂ acts mainly through EP2 and EP4 receptors to elevate cAMP, antagonists targeting EP2 (AH6809) and EP4 (L-161982) were used [22, 23]. Pretreatment with the EP4 antagonist but not the EP2 antagonist abolished EPG-induced decidual marker expression (Fig. 1E). Conversely, the EP4 agonist CAY10598 alone was sufficient to induce FOXO1, IGFBP1, and PRL expression, as well as morphological changes typical of decidualization (Fig. 1F; Fig. S1C, D). These effects were further amplified by co-treatment with steroid hormones (Fig. 1F). Immunofluorescence staining revealed that EP4 localized to both the primary cilia (stained by ARL13B) and the plasma membrane in ESCs isolated from human endometrium, as well as in ESCs within human and murine endometrial tissues, whereas EP2 was restricted to the plasma membrane (Fig. 1G–I; Fig. S1E). Within the endometria, primary cilia were present exclusively in ESCs, but absent from surface and glandular epithelial cells (Fig. S1F, G).

Because Hedgehog (Hh) signaling has been reported to promote decidualization in mice [24]. We next examined whether it also regulates decidualization in human ESCs. Smoothened, the key receptor mediating Hh signaling, was detected at the primary cilium (Fig. S2A). Upon stimulation with Hh ligands, including Indian hedgehog (IHH), Sonic hedgehog (SHH), and Desert hedgehog (DHH), Smoothened translocated to the cilium and activated downstream GLI transcriptional targets [25]. Activation of Hedgehog signaling using recombinant SHH or a Smoothened agonist did not induce expression of decidual markers FOXO1 or IGFBP1 (Fig. S2B). When combined with EPC, SHH slightly increased IGFBP1 levels but did not affect FOXO1 expression (Fig. S2C). Although the Smoothened agonist activated downstream targets such as *GLI1* and *PTCH1*, it failed to promote decidualization (Fig. S2D–F). SHH protein was detected in mouse endometrial tissue but was nearly undetectable in human ESCs, and the active N-terminal SHH fragment was absent (Fig. S2G, H). Consistently, SHH also failed to induce IGFBP1 and FOXO1 expression (Fig. S2I). These findings suggest that primary cilia may provide a platform for EP4-mediated PGE₂ signaling during decidualization, while Hedgehog signaling does not play any significant role in human ESCs.

### Ciliary EP4 signaling drives PGE₂-induced decidualization via the PKA-CREB pathway

**Fig. 2:**
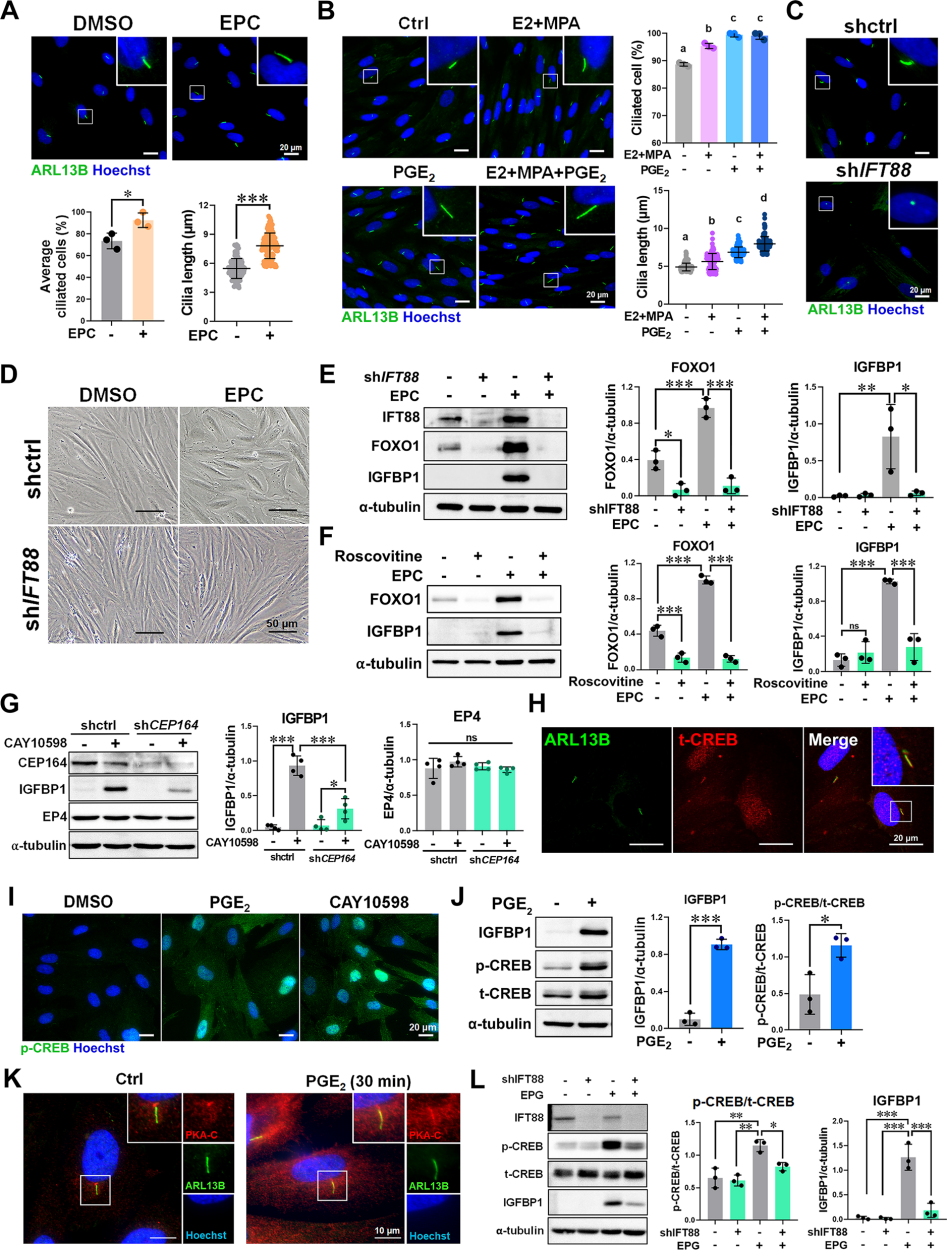
Ciliary PGE_2_/EP4 signaling regulates the expression of decidualization markers.** A** Immunofluorescence images and analysis of primary cilia (anti-ARL13B antibody, green) in ESCs treated with EPC or DMSO for 6 days (n = 3 biological replicates). Scale bar = 20 μm. **B** Immunofluorescence images and quantification of ciliogenesis in ESCs treated with DMSO (control) and E2 (10 nM) combined with MPA (1 μM) in the presence of PGE_2_ (1 μM) or not under serum-free conditions (n = 3 biological replicates, A) for 4 days. Scale bar = 20 µm. Groups with different letters (a, b, c, d) are significantly different (p < 0.05). **C** Immunofluorescence images of *IFT88*-knockdown (sh*IFT88*) and control (shctrl) ESCs. Scale bar = 20 μm. **D** Bright-field images of shctrl and sh*IFT88* ESCs treated with EPC or DMSO for 6 days. Scale bar = 50 μm. **E** Western blot images and quantification of proteins post-EPC or DMSO treatment for 6 days (n = 3 biological replicates). **F** Western blot images and quantified results of IGFBP1 and FOXO1 in ESCs treated with 20 μM roscovitine in the presence of EPC or DMSO for 48 h (n = 3 biological replicates). **G** Western blot images and quantification of IGFBP1 and EP4 in shctrl and sh*CEP164* ESCs treated with CAY10598 (1 μM) for 4 days (n = 4 biological replicates). **H** Localization of total CREB (t-CREB, red) and ARL13B (green) in ESCs, with nuclei stained using Hoechst (blue). **I** Immunofluorescence images showing the localization of p-CREB (green) in ESCs after treatment with PGE_2_ (10 μM) or CAY10598 (10 μM) for 4 h. Scale bar = 20 μm. **J** Western blot images and quantification of IGFBP1, phosphorylated CREB (p-CREB), and total CREB (t-CREB) in ESCs treated with PGE_2_ (10 μM) for 4 h (n = 3 biological replicates). Nuclei were stained with Hoechst (blue). **K** Immunofluorescence images showing the localization of the PKA catalytic subunit (PKA-C, red) and primary cilia (ARL13B, green) in ESCs treated with PGE_2_ (10 μM) or DMSO for 30 min. Scale bar = 10 μm. **L** Western blot images and quantification of proteins in shctrl and sh*IFT88* ESCs treated with EPG cocktail for 4 days (n = 3 biological replicates). *p ≤ 0.05, **p ≤ 0.01, ***p ≤ 0.001 by two-way ANOVA with Tukey’s multiple test (panels **B**, **E**, **F**, **G**, and **L**), or Student’s *t*-test (panel **A** and **J**)

We examined the ciliogenesis in ESCs in response to decidualization stimuli. EPC treatment significantly increased both the proportion of ciliated ESCs and the average ciliary length (Fig. 2A). PGE₂ alone promoted ciliogenesis, and this effect was further enhanced by co-treatment with E2 and MPA (Fig. 2B). To determine whether primary cilia are required for decidualization, we disrupted ciliogenesis in ESCs by genetic ablation of *IFT88* or *CEP164*, both of which impaired cilia formation (Fig. 2C; Fig. S3A-C). Loss of primary cilia significantly attenuated decidualization (Fig. 2D, E; Fig. S3D, E). Similarly, treatment with roscovitine, a cyclin-dependent kinase 5 inhibitor that suppresses cilium formation [26], impaired both ciliogenesis and EPC-induced decidualization markers (Fig. 2F; Fig. S3F), indicating that primary ciliary signaling is indispensable for decidualization. Because PGE₂ is known to activate the ciliary cAMP signalosome through EP receptors [27], we next examined the role of ciliary EP4 in decidualization. Knockdown of *CEP164* markedly reduced EP4 agonist CAY10598-induced IGFBP1 expression, despite unaltered overall EP4 levels (Fig. 2G). Together, these results demonstrate that primary cilia are essential for ESCs’ decidualization and may serve as a functional marker for uterine receptivity.

Bioinformatic analysis identified a cAMP-responsive element in the IGFBP1 promoter that can be bound by phosphorylated cAMP-response element binding protein (p-CREB) (Fig. S3G). Consistently, CREB was detected at the primary cilium (Fig. 2H), and PGE₂ treatment triggered CREB phosphorylation and nuclear translocation (Fig. 2I, J), coinciding with IGFBP1 induction (Fig. 2J; Fig. S3H). The EP4 agonist mimicked this effect (Fig. 2J; Fig. S3I). Notably, the PKA catalytic subunit was enriched at primary cilia, and p-CREB rapidly translocated to the nucleus following short-term PGE₂ treatment (Fig. 2K; Fig. S3J). Similarly, short-term treatment with PGE_2_ or EP4 agonist also induced FOXO1 expression (Fig. S3K, L) and nuclear translocation (Fig. S3M). Knockdown of IFT88 to inhibit primary cilia formation abolished EPG-induced CREB phosphorylation and IGFBP1 upregulation (Fig. 2L). These findings demonstrate that ciliary EP4 signaling activates the PKA-pCREB axis to drive IGFBP1 expression and promote decidualization.

### Elevated TGF-β1 in women with endometriosis impairs cilia-mediated decidualization

**Fig. 3:**
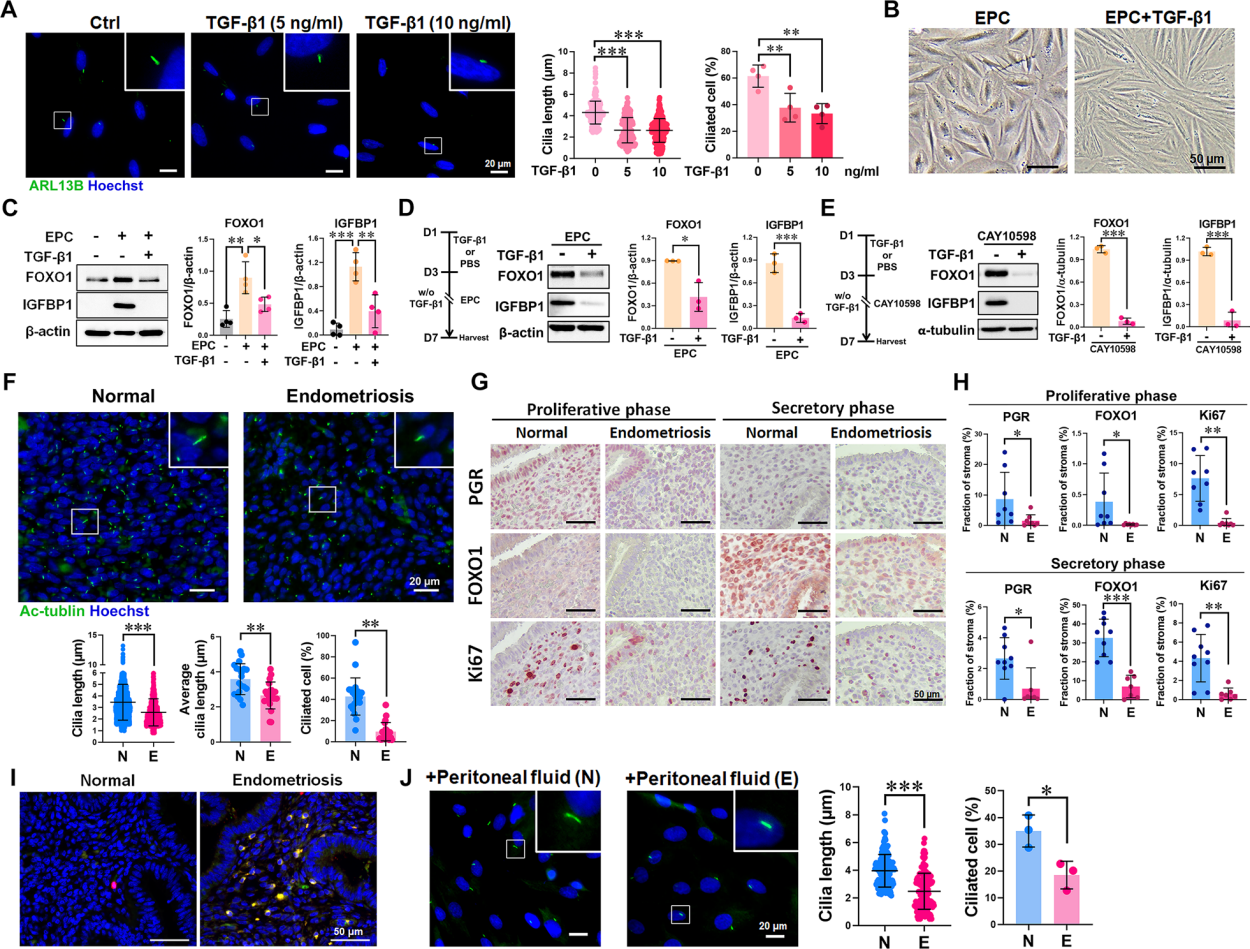
Elevated TGF-β1 disrupts ciliogenesis and decidualization in endometriosis. **A** Representative images and quantitative results of ESC primary cilia (ARL13B, green) after 5 or 10 ng/ml TGF-β1 (n = 4 biological replicates). Cells treated with PBS for 48 h served as controls (Ctrl). Scale bar = 20 μm. **B** and **C** ESCs were treated with vehicle (Ctrl), EPC, and EPC with TGF-β1 (10 ng/ml) for 6 days. Representative bright-field images show the morphology of ESCs. Scale bar = 50 μm (**B**). Representative Western blot images and quantified results of FOXO1 and IGFBP1 in ESCs (n = 4 biological replicates, **C**). **D** and **E** Schematic diagrams showing the experimental procedures and Western blot analysis of FOXO1 and IGFBP1 in ESCs pretreated with TGF-β1 (10 ng/ml) for 48 h, followed by EPC (n = 3 biological replicates, **D**) or 1 μM CAY10598 (n = 3 biological replicates, **E**) treatment for 4 days. **F** Immunofluorescence analysis of primary cilia in secretory-phase endometria from women with (**E**, n = 20) and without (**N**, n = 20) endometriosis. Normal specimens are defined as those without endometriosis. Primary cilia and nuclei were stained with anti-Ac-tubulin (green) and Hoechst (blue). Scale bar = 20 μm. **G** and **H** IHC images (**G**) and quantification (**H**) of PGR, FOXO1, and Ki67 expression in proliferative and secretory-phase endometria from normal (**N**) and endometriosis (**E**) patients. Positive stromal signals were quantified. Proliferative-phase normal: n = 8; endometriosis: n = 8; secretory-phase normal: n = 9; endometriosis: n = 7. Scale bar = 50 μm. **I** Immunofluorescence of endometrial macrophages (red) co-stained with TGF-β1 (green) and nuclei (Hoechst, blue). Scale bar = 50 μm. **J** Immunofluorescence analysis of primary cilia (ARL13B, green) in ESCs treated with peritoneal fluid (diluted 100 ×) from normal (N = 5) or endometriosis (E = 5) patients. Ciliogenesis was quantified after 48 h (n = 3 biological replicates). Scale bar = 20 μm. *p ≤ 0.05, **p ≤ 0.01, ***p ≤ 0.001 by Student’s t-test, Mann–Whitney *U* test, or one-way ANOVA with Tukey’s test (for three groups)

Ciliogenesis is regulated by cytokines, and pro-inflammatory factors are frequently elevated in gynecological disorders such as endometriosis [28–30]. In particular, TGF-β1, interleukin-6 (IL-6), and tumor necrosis factor-α (TNF-α) are markedly increased in endometriosis and have been implicated in disease pathophysiology [18, 31, 32]. TGF-β1 significantly reduced both cilia length and the proportion of ciliated ESCs (Fig. 3A), whereas IL-6 and TNF-α had minimal effects (Fig. S4A, B). TGF-β1 also suppressed EPC-induced IGFBP1 and FOXO1 expression, and FOXO1 nuclear localization (Fig. 3B, C; Fig. S5A, B). In contrast, IL-6 and TNF-α did not influence EPG-induced IGFBP1 expression (Fig. S5C). Pretreatment with TGF-β1 before EPC or EP4 agonist stimulation decreased IGFBP1 and FOXO1 induction, without altering EP4 expression (Fig. 3D, E; Fig. S5D). These results suggest that TGF-β1 impairs primary ciliogenesis and thereby attenuates cilia-dependent decidual signaling.

Given the established link between endometriosis and infertility [33, 34], we examined endometrial cilia in secretory-phase tissues from women with (n = 20) and without (n = 20) endometriosis. Compared to controls, endometriosis samples showed shorter cilia and fewer ciliated ESCs (Fig. 3F), findings consistent across early, mid, and late secretory phases (Figure S5E, F), and most pronounced in advanced disease (Fig. S5G). Immunostaining further revealed reduced expression of decidual markers, including PGR, FOXO1, and Ki67 (Fig. 3G, H). ESCs isolated from endometriosis samples showed reduced PRL and IGFBP1 expression in response to decidual cues (Fig. S5H), indicating a compromised decidual response. Given the functional effects of TGF-β1 on ESCs, we next assessed its levels in uterine and peritoneal fluid from endometriosis patients. Indeed, uterine fluid and peritoneal fluid from women with endometriosis showed significantly higher TGF-β1 concentrations compared to controls (Fig. S5I, J). Immunofluorescence analysis revealed increased TGF-β1-positive macrophages in the endometrial tissues of women with endometriosis (Fig. 3I; Fig. S5K), suggesting that endometrial immune cells contribute to local TGF-β1 production. Additionally, ESCs treated with peritoneal fluid from endometriosis patients showed impaired ciliogenesis, with shorter cilia and a reduced proportion of ciliated cells (Fig. 3J). Peritoneal TGF-β1 concentrations were negatively correlated with cilia length and percentage of ciliated cells (Fig. S5L), indicating that TGF-β1 present in peritoneal fluid may reach the endometrial environment to impair ciliogenesis. Together, these results support a model that an elevated level of TGF-β1 suppresses ciliogenesis and compromises decidualization in women with endometriosis.

### TGF-β1 suppresses ciliogenesis through the regulation of COUP-TFII and KIF3B

**Fig. 4:**
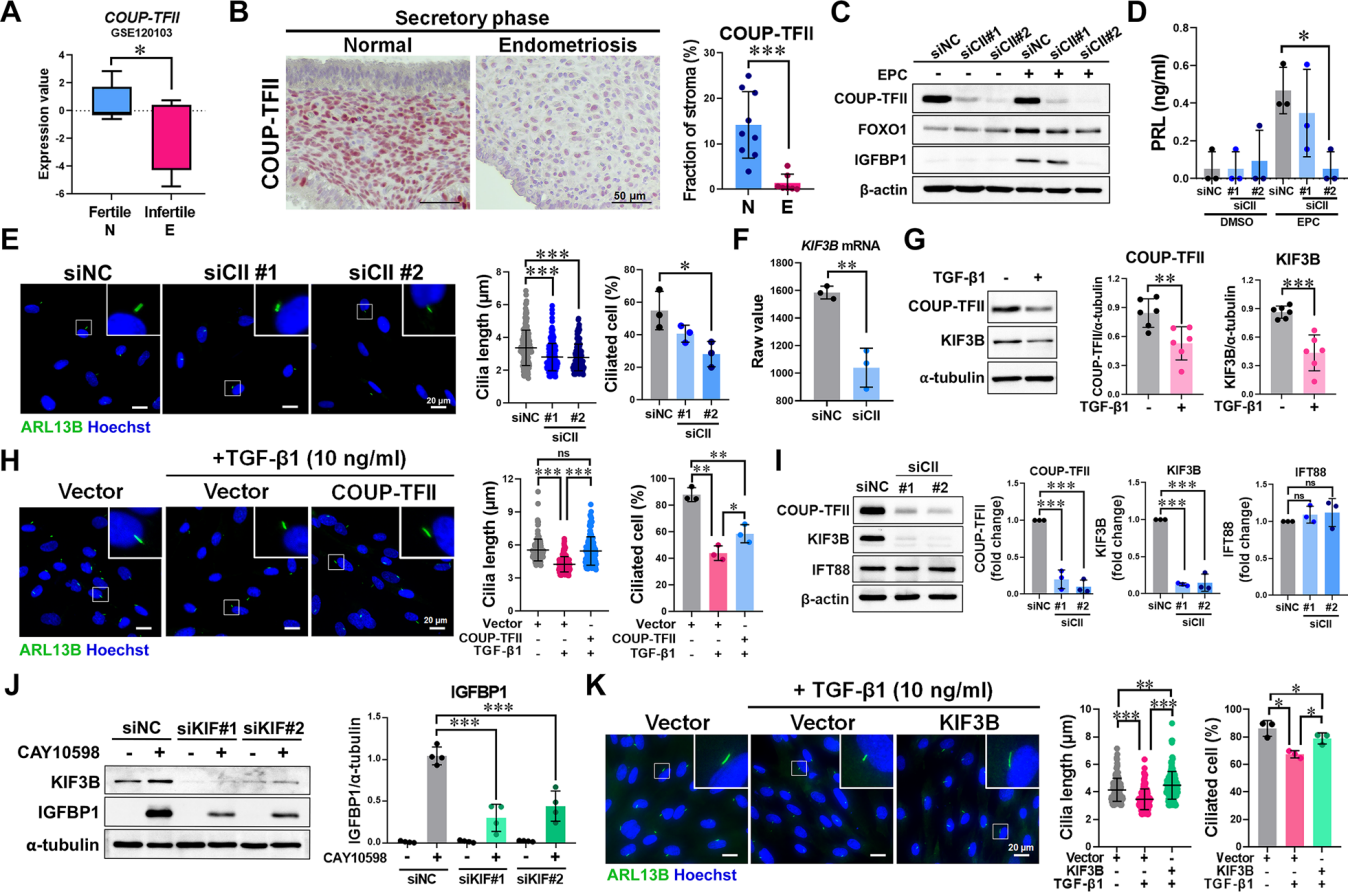
TGF-β1 suppresses ciliogenesis by downregulating COUP-TFII and KIF3B. **A** COUP-TFII expression in the endometria of fertile women without endometriosis (n = 9) and infertile women with endometriosis (n = 9) from the GEO dataset GSE120103. **B** The IHC staining and quantification of COUP-TFII in endometrial stroma from normal women (n = 9) and women with endometriosis (n = 7). **C** Western blot analysis of proteins in *COUP-TFII*-knockdown (siCII) or control (siNC) ESCs treated with EPC or DMSO for 6 days. **D** ELISA of PRL levels in media from ESCs treated with EPC or DMSO for 6 days (n = 3 biological replicates). **E** Immunofluorescence and quantification of ciliogenesis in ESCs cultured in serum-free medium for 48 h (n = 3 biological replicates). Scale bar = 20 μm. **F** *KIF3B* mRNA levels in siCII (n = 3 biological replicates) and siNC ESCs (n = 3 biological replicates) from dataset GSE107469. **G** Western blotting and quantification of COUP-TFII and KIF3B in ESCs after TGF-β1 (10 ng/ml) treatment for 48 h (n = 6 biological replicates). **H** Representative images and quantitative results of primary cilia (ARL13B, green) in ESCs transfected with empty vector (pcDNA) or *COUP-TFII*-Flag plasmid treated with or without 10 ng/ml TGF-β1 (n = 3 biological replicates). Nuclei were stained with Hoechst (blue). Scale bar = 20 μm. **I** Western blotting and quantification of proteins in siCII and siNC ESCs (n = 3 biological replicates). **J** Western blotting and quantification of IGFBP1 in siKIF and siNC ESCs treated with 1 μM CAY10598 or vehicle for 4 days (n = 4 biological replicates). **K** Representative images and quantitative results of primary cilia (ARL13B, green) in ESCs transfected with empty vector or *KIF3B* plasmid treated with or without 10 ng/ml TGF-β1 (n = 3 biological replicates). * p ≤ 0.05, ** p ≤ 0.01, ***p ≤ 0.001 by Mann–Whitney *U* test, Student’s *t*-test (for two groups), one-way ANOVA with Tukey’s multiple test (for three or multiple groups)

To explore the mechanism by which TGF-β1 impairs ciliogenesis, we analyzed microarray data (GSE120103) from endometrial tissues of fertile women and infertile women with endometriosis [35]. Among genes downregulated in the infertile group, 10 overlapped with known decidualization-associated genes [36] (Fig. S6A, B). We focused on COUP-TFII (also known as NR2F2), which is known to be repressed by TGF-β1 in endometriotic stromal cells [37]. Public datasets confirmed reduced COUP-TFII expression in endometria from infertile women with endometriosis (Fig. 4A). IHC on our patient samples validated lower COUP-TFII levels in secretory-phase endometria of women with endometriosis compared to healthy controls (Fig. 4B). To determine COUP-TFII’s role in ciliogenesis, we knocked down its expression in ESCs. Loss of COUP-TFII impaired ciliogenesis, shortened ciliary length, and suppressed decidual markers, including FOXO1, IGFBP1, and PRL (Fig. 4C–E). To identify downstream targets, we intersected a previously published *COUP-TFII*-knockdown transcriptome with a list of ciliogenesis-related genes, identifying nine candidate genes (Fig. S6C). KIF3B was the most significantly downregulated gene in this list (Fig. 4F). We further found that COUP-TFII and KIF3B mRNA levels were reduced in eutopic endometrial tissues from women with endometriosis (Fig. S6D). TGF-β1 treatment downregulated COUP-TFII and KIF3B in ESCs (Fig. 4G), while overexpression of COUP-TFII restored cilia length and increased the percentage of ciliated cells in the presence of TGF-β1 (Fig. 4H). KIF3B expression was diminished in *COUP-TFII-*knockdown cells (Fig. 4I) and increased upon COUP-TFII overexpression (Fig. S6E). Functionally, knockdown of *KIF3B* reduced ciliogenesis (Fig. S6F, G) and impaired decidualization responses, as indicated by reduced IGFBP1 expression following treatment with either EP4 agonist or EPG medium (Fig. 4J; Fig. S6H, I). In contrast, overexpression of KIF3B not only rescued COUP-TFII knockdown-suppressed ciliogenesis but also restored TGF-β1-inhibited ciliary elongation (Fig. S6J, K; Fig. 4K). Together, these data demonstrate that TGF-β1 disrupts ESC ciliogenesis and decidualization by downregulating COUP-TFII and its downstream effector KIF3B.

### Uterine TGF-β1 impairs embryo implantation in vivo

**Fig. 5:**
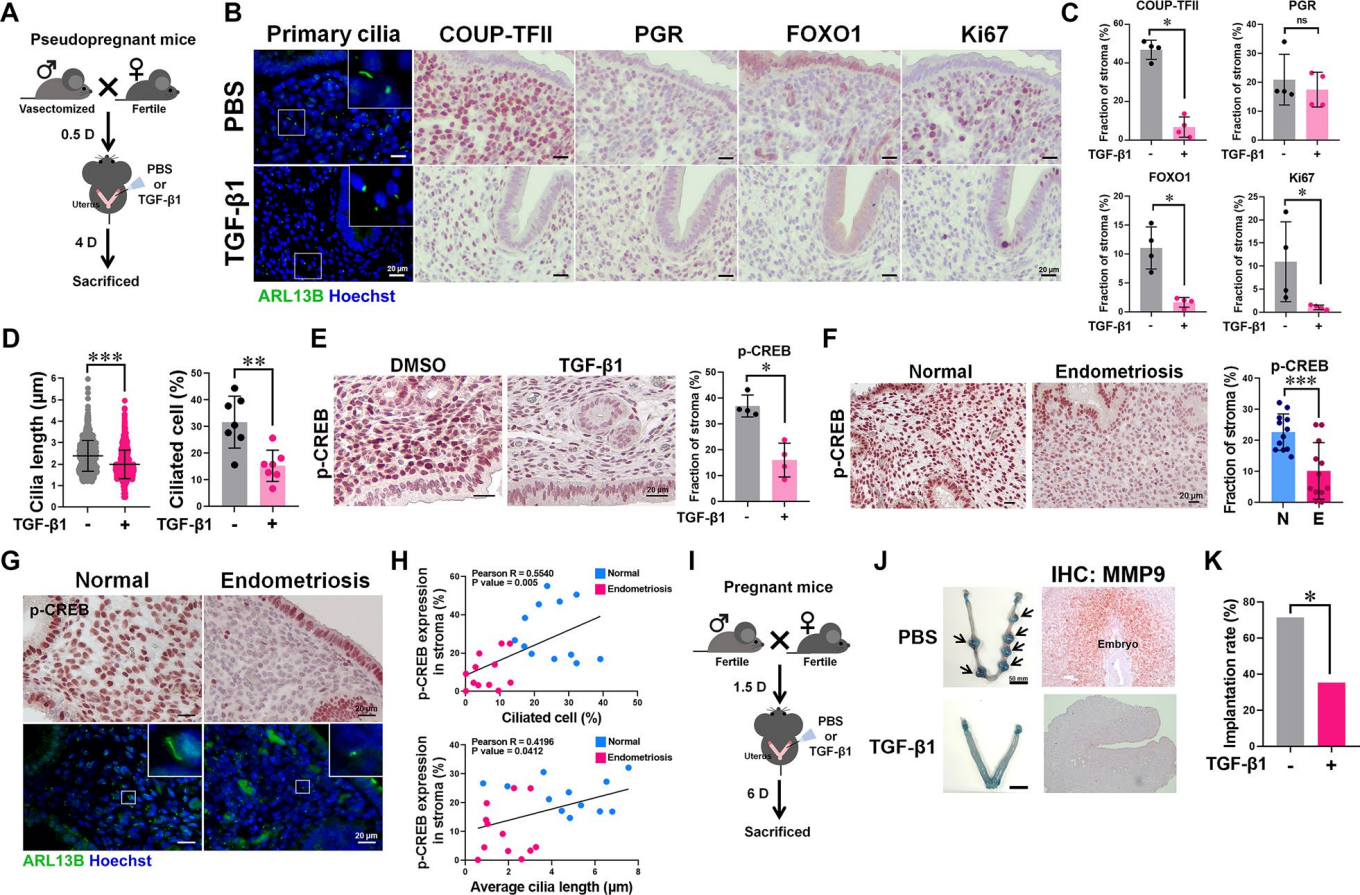
TGF-β1 impairs uterine decidualization and embryo implantation. **A** Schematic illustration of the procedure for intrauterine injection of TGF-β1 in a 4.5 dpc female mouse model.** B** (**B**) Representative immunofluorescence and IHC images of 4.5 dpc mouse uteri treated with PBS or TGF-β1, showing primary cilia (ARL13B, green) and the expression of COUP-TFII, PGR, FOXO1, and Ki67. Primary cilia were detected with an anti-ARL13B antibody, and nuclei were counterstained with Hoechst in the immunofluorescence images. Scale bar = 20 μm. **C** Quantification of COUP-TFII, PGR, FOXO1, and Ki67 staining is shown in (**B**) (n = 4 per group). **D** Quantified ciliogenesis in the endometria of 4.5 dpc mice treated with PBS (n = 7) or TGF-β1 (n = 7). **E** IHC staining and quantitative results of p-CREB in 4.5 dpc mouse uteri (n = 4 per group). Scale bar = 20 μm. **F** The IHC staining and quantitative results of p-CREB in endometria from secretory-phase women without (N, n = 12) or with endometriosis (E, n = 12). Scale bar = 20 μm. **G** The IHC staining of p-CREB and immunofluorescence staining of ARL13B (green) in serial sections of secretory phase human endometria. Nuclei were stained with Hoechst in the immunofluorescence images. Scale bar = 20 μm. **H** Pearson correlation analysis between p-CREB expression, percentage of ciliated cells, and cilia length in the endometrial stroma region. A total of 24 endometrial specimens from women in the secretory phase were quantified (normal: n = 12, endometriosis: n = 12). **I** Schematic illustration of the procedure for a 7.5 dpc pregnant mouse model. **J** Representative images showing the embryo implantation site (stained by Evans blue) and the IHC staining of MMP9 in decidualized stromal cells surrounding the embryo. **K** Percentage of mice with implants after treatment with PBS (n = 21) or TGF-β1 (n = 17) on 7.5 dpc. *p ≤ 0.05, **p ≤ 0.01, ***p ≤ 0.001 by Mann–Whitney *U* test, Student’s *t*-test (for two groups), or Chi-square test (panel **K**)

To test whether elevated TGF-β1 levels impair endometrial function and embryo implantation, we performed intrauterine injections of TGF-β1 in pseudopregnant mice at 0.5 days post coitum (dpc) (Fig. 5A). TGF-β1 administration reduced the expression of COUP-TFII, FOXO1, and Ki67 (Fig. 5B, C), shortened cilia, and decreased the percentage of ciliated stromal cells (Fig. 5D), suggesting impaired uterine decidualization. Stromal p-CREB levels were lower in TGF-β1-treated mice (Fig. 5E), resembling the phenotype observed in women with endometriosis (Fig. 5F). In human endometrial samples, p-CREB levels positively correlated with cilia length and the percentage of ciliated stromal cells (Fig. 5G, H). We then assessed implantation rate at 7.5 dpc (Fig. 5I). In PBS-treated control mice, implanted embryos were surrounded by MMP9-positive decidualized stromal cells. In contrast, embryos were largely absent and MMP9-negative in the endometria of TGF-β1-treated mice (Fig. 5J). The implantation rate in the TGF-β1 group was significantly lower than that in controls (35.3% vs. 71.4%; Fig. 5K). Histological analysis confirmed no overt abnormalities in the embryos, suggesting that the reduced implantation was due to uterine dysfunction rather than embryonic defects (Fig. S7).

### Blocking TGF-β signaling restores ciliogenesis and improves implantation

**Fig. 6:**
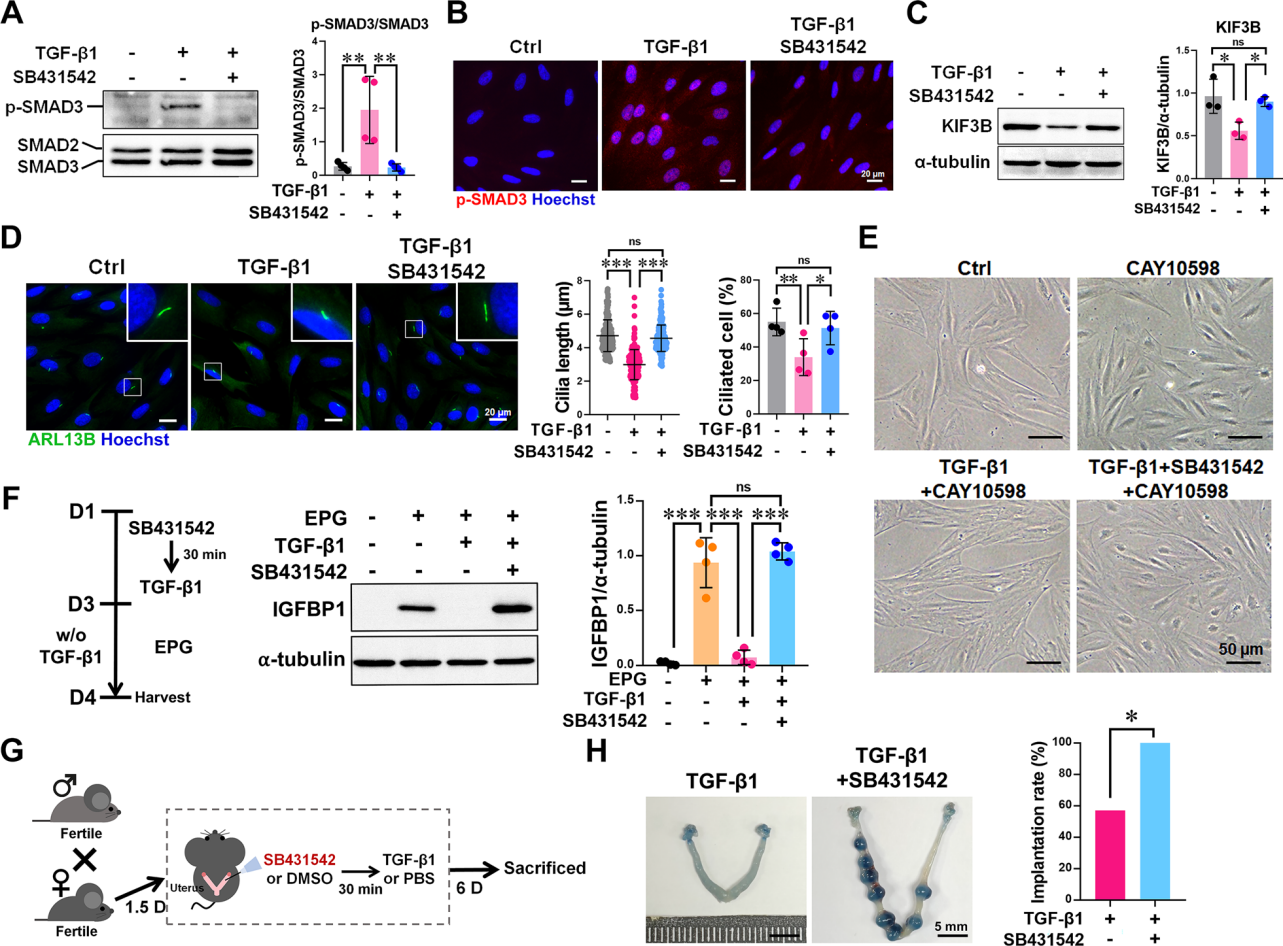
Restoration of ciliogenesis, decidualization, and embryo implantation by blocking TGF-β signaling. **A** and **B** ESCs were pretreated with 10 μM SB431542 for 30 min before treatment with 10 ng/ml TGF-β1 and incubated for 24 h. Western blot images and quantification of p-SMAD3 and SMAD2/3 in ESCs (n = 4 biological replicates,** A**). Immunofluorescence images of p-SMAD3 (red) in ESCs with nuclei stained by Hoechst (blue). Scale bar = 20 μm (**B**). **C** and **D** ESCs were pretreated with 10 μM SB431542 for 30 min before treatment with 10 ng/ml TGF-β1 and incubated for 48 h. Western blot images and quantification of KIF3B in ESCs (n = 3 biological replicates, **C**). Immunofluorescence images and quantification of ciliogenesis in ESCs (n = 4 biological replicates). Scale bar = 20 μm (**D**). **E** Bright-field images of ESCs pretreated with 10 μM SB431542 and TGF-β1 (10 ng/ml), followed by 1 μM CAY10598 treatment for 4 days. Scale bar = 50 μm. **F** Schematic diagram of experimental procedures: ESCs were pretreated with 10 μM SB431542 for 30 min before adding 10 ng/ml TGF-β1 and incubating for 48 h, followed by EPG medium treatment for 24 h. Western blot images and quantification of IGFBP1 in ESCs (n = 4 biological replicates). **G** Schematic of the intrauterine injection procedure for SB431542 and TGF-β1 in a pregnant mouse model. **H** Images showing embryo implantation sites (stained by Evans blue) and the percentage of mice with implants after treatment with TGF-β1 (n = 7) or TGF-β1 with SB431542 (n = 6) on 7.5 dpc. *p ≤ 0.05, **p ≤ 0.01, ***p ≤ 0.001 by one-way ANOVA with Tukey’s multiple test (for three or multiple groups), or Chi-square test (panel **H**)

Given the inhibitory effects of TGF-β1 on ESC ciliogenesis and decidualization, we investigated whether blocking TGF-β signaling could reverse these impairments. ESCs pretreated with the TGF-β receptor I inhibitor SB431542 showed abolished SMAD3 phosphorylation and nuclear translocation upon TGF-β1 treatment (Fig. 6A, B), restoration of KIF3B expression, and recovery of primary cilia formation (Fig. 6C, D). SB431542 also restored TGF-β-inhibited decidualization in ESCs treated with EP4 agonist or EPG medium, as indicated by increased IGFBP1 expression and characteristic morphological changes (Fig. 6E, F). To validate this in vivo, we pre-injected SB431542 into the uteri of mice 30 min before TGF-β1 administration and assessed implantation at 7.5 dpc (Fig. 6G). SB431542 significantly rescued the implantation rate compared to TGF-β1 alone (100% vs. 57.1%; Fig. 6H). These findings demonstrate that pharmacological inhibition of TGF-β signaling restores primary cilia-dependent PGE_2_ signaling and decidualization, ultimately improving endometrial receptivity and embryo implantation.

### Endometrial ciliogenesis is positively correlated with IVF-ET outcomes

**Fig. 7:**
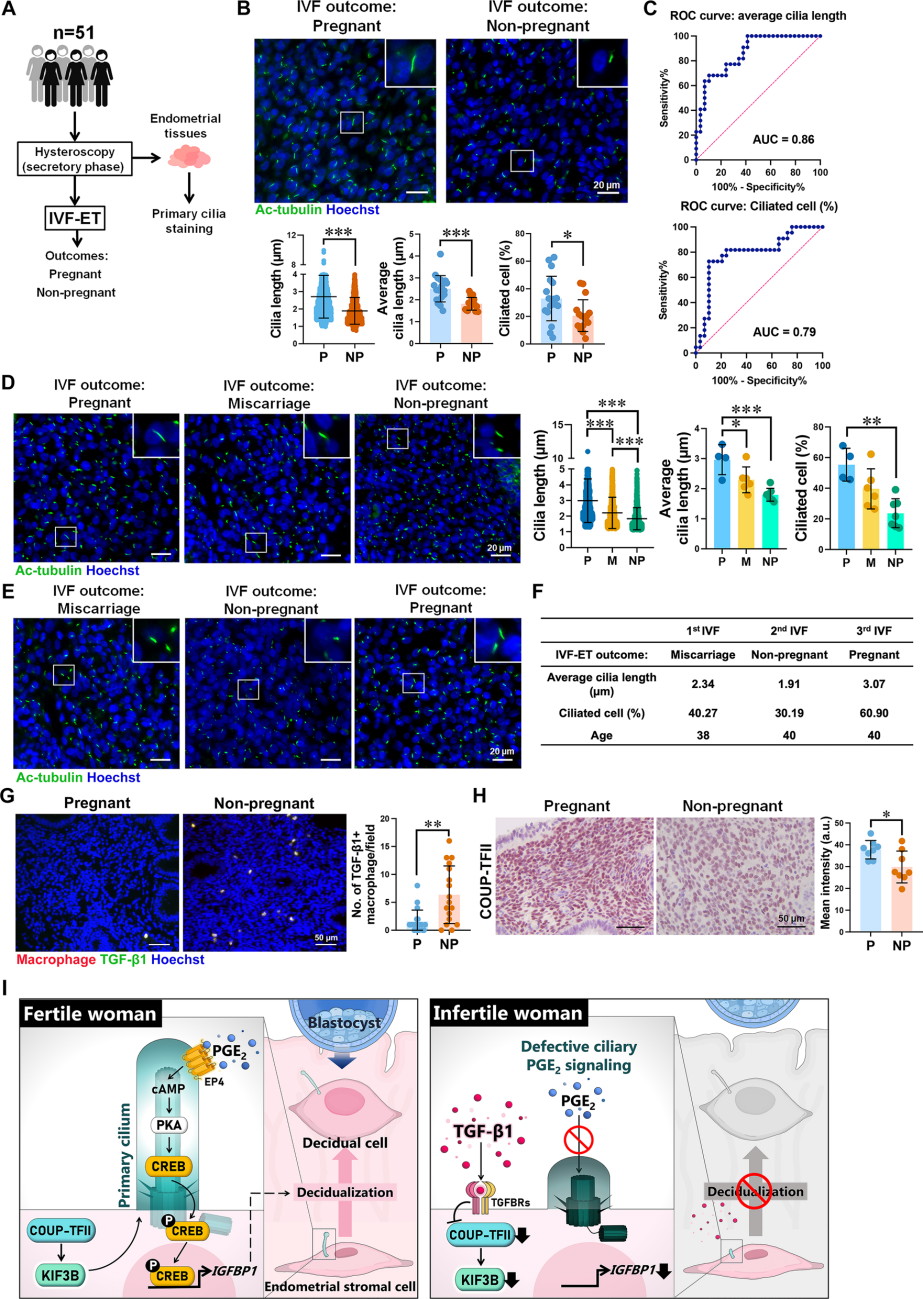
Primary cilia in ESCs are essential for uterine receptivity and decidualization. **A** Diagram of endometrial tissue collection from 51 infertile women during the secretory phase via hysteroscopy before IVF-ET. **B** Immunofluorescence images and quantitative analysis of primary cilia in secretory-phase endometrial tissues from women undergoing IVF-ET. The outcomes of IVF-ET are categorized as pregnant (P, n = 29) and non-pregnant (NP, n = 22). Primary cilia and nuclei are stained with anti-acetyl-tubulin (Ac-tubulin, green) and Hoechst (blue). Scale bar = 20 μm. **C** ROC curves illustrating the diagnostic accuracy of average cilia length (AUC = 0.86) and percentage of ciliated cells (AUC = 0.79) in predicting IVF-ET outcomes. **D** Immunofluorescence images and quantitative analysis of primary cilia in secretory endometrium from women with IVF-ET outcomes: P (n = 4), miscarriage (M, n = 6), NP (n = 7). **E** and **F** Immunofluorescence staining of primary cilia in the endometrium of a woman who underwent three IVF-ET cycles, categorized by IVF outcome. Anti-Ac-tubulin antibody was used to stain primary cilia (green), and nuclei were stained with Hoechst (blue). Scale bar = 20 µm (**E**). The quantitative analysis of average cilia length, percentage of ciliated cells, and age at IVF-ET (**F**). **G** Representative immunofluorescence images (left panel) and quantified results (right panel) show TGF-β1 signals (green) and macrophages (red) within the endometrium of women undergoing IVF-ET. The number of TGF-β1-positive macrophages was quantified in pregnant women (n = 19) and non-pregnant women (n = 17). Scale bar = 50 μm. **H** IHC staining (left panel) and quantitative results (right panel) of COUP-TFII in the endometrium of women undergoing IVF-ET. Pregnant women (n = 8) and non-pregnant women (n = 8) were included. Scale bar = 50 μm. **I** Schematic diagram of the working model. *p ≤ 0.05, **p ≤ 0.01, ***p ≤ 0.001 by Mann–Whitney *U* test, Student’s *t*-test, or one-way ANOVA with Tukey’s multiple test

Finally, to assess the medical significance, we retrospectively analyzed samples from 51 women undergoing IVF-ET (Fig. 7A). No significant differences were observed between pregnant (n = 29) and non-pregnant (n = 22) groups in maternal age, BMI, gravidity, and endometrial thickness, while anti-Mullerian Hormone (AMH) showed a weak association (Supplementary Tables S1 and S2). In contrast, women who failed to conceive exhibited fewer and shorter primary cilia in their ESCs compared to those who became pregnant (Fig. 7B). To evaluate the diagnostic performance of primary cilia-related parameters in predicting IVF-ET outcomes, we performed receiver operating characteristic curve analyses (Fig. 7C). Using cilia length of 2.403 μm (AUC = 0.86) as a cutoff value yielded a sensitivity of 100% and specificity of 58.62%, while a ciliated cell percentage of 22.51% (AUC = 0.79) showed 72.73% sensitivity and 89.66% specificity (Fig. 7C). In IVF-ET patients undergoing the endometrial receptivity assay (n = 17), cilia were shorter and less prevalent in women who failed to conceive, whereas women who experienced early miscarriage had similar ciliated cell percentages but shorter cilia compared to those with successful pregnancies (Fig. 7D). Notably, in a longitudinal case of repeated IVF cycles, cilia length positively correlated with pregnancy success (Fig. 7E and F), suggesting a link between endometrial primary cilia and uterine receptivity, providing additional support for the link between cilia integrity and uterine receptivity. Furthermore, non-pregnant women exhibited a higher number of TGF-β1-positive macrophages and reduced COUP-TFII expression in the endometrium (Fig. 7G and H). Collectively, these results indicate that functional primary cilia contribute to endometrial receptivity and improved IVF-ET outcomes, and that increased TGF-β1 signaling may lead to implantation failure through ciliary dysfunction (Fig. 7I).

## Discussion

A receptive uterus is a prerequisite for the initiation of pregnancy, and decidualization is the first step in transforming a non-receptive uterus into a receptive one. Here, we show that primary cilium-mediated PGE_2_ signaling is required for this transition, and that aberrantly elevated TGF-β1 impairs decidualization by disrupting ciliogenesis. Our data delineate a novel mechanism responsible for human uterine decidualization and reveal the cause of reduced uterine receptivity, which may provide a useful means to improve pregnancy rates for women undergoing IVF or suffering from endometriosis.

The primary cilium, as a signaling hub, regulates diverse biological processes that mediate communication between the extracellular and intracellular compartments. In human uterine ESCs, primary cilia elongate after ovulation, likely stimulated by estrogen, progesterone, and PGE_2_, as combined treatment with these three factors shows an additive effect on ciliogenesis. Our findings highlight the essential role of primary cilia in endometrial receptivity, with genetic or pharmacological inhibition leading to decidualization failure. This finding may explain the observation that women who fail to conceive after IVF-ET have fewer and shorter primary cilia in their uterine ESCs.

While the role of primary cilia in decidualization is established, the precise downstream pathways remain to be elucidated. In murine models, Hedgehog signaling has been implicated in decidualization, but its role in human ESCs remains less defined. Ablation of uterine *Ihh* suppresses decidualization in mice [38], and Shh-mediated Hedgehog signaling activation increases the expression of PRL family members *Prl8a2* and *Prl3c* in murine ESCs [24]. While Hedgehog signaling appears critical for decidualization in mice, its role in humans is less clear. Although increased expression of IHH and GLI1 has been observed in human endometrial epithelial cells during the secretory phase [39], the effect of Hedgehog signaling in human endometrial decidualization remains uncharacterized. Our data demonstrate that, unlike in mice, Hedgehog signaling is dispensable for human ESC decidualization, as neither recombinant SHH nor Smoothened agonist significantly induced decidual markers. Instead, our findings indicate that PGE_2_ signaling is the primary driver of decidualization in human ESCs. Specifically, PGE_2_ binds to ciliary EP4 and activates PKA-mediated CREB phosphorylation, phosphorylated CREB then translocates to the nucleus and initiates the transcription of decidual marker genes. Although PGE_2_ can signal through both EP2 and EP4 to activate PKA, our data showed that only blocking EP4-mediated signaling abolished PGE_2_’s action, indicating the decidualization signal was predominantly transmitted via the EP4 receptor. Immunofluorescence staining showed that EP4 receptors reside on both the primary cilia and plasma membrane, whereas EP2 is restricted to the plasma membrane. Mechanistically, by disrupting primary cilia formation through genetic knockdown and pharmacological inhibition, we demonstrate, for the first time, that decidualization of human endometrial cells is driven by ciliary PGE_2_ signaling. This spatial compartmentalization explains why EP4, rather than EP2, is the essential mediator of this process. Our findings echo the landmark discovery that prostaglandins initiate blastocyst implantation [40], providing a long-awaited mechanistic explanation for this 50-year-old puzzle. Collectively, our data indicate that the primary cilium is a critical organelle for the PGE_2_/EP4/PKA/CREB signaling in decidualization, suggesting that ciliary dysfunction may be a key factor in impaired decidualization.

One of the most intriguing findings in this study is that women who fail to conceive after IVF-ET have fewer and shorter primary cilia in ESCs. Our data show that treatment with TGF-β1 inhibits primary cilia formation in ESCs, which prevents PGE_2_-induced ESC decidualization. Mechanistically, we identify a novel regulatory axis in which TGF-β1 suppresses COUP-TFII, leading to downregulation of the ciliogenic gene KIF3B and impaired decidualization. KIF3B is a critical subunit of the Kinesin-2 motor complex, which is responsible for intraflagellar anterograde transport of building blocks and receptors to maintain the steady-state length of the cilium. Ablation or inhibition of KIF3B will result in shortening or disappearance of primary cilia because the natural disassemble at the tip of the cilia. We found that COUP-TFII is required for endometrial primary cilia formation by stimulating the expression of the ciliogenic gene, *KIF3B*. Knockdown of either *COUP-TFII* or *KIF3B* results in reduced ciliogenesis and impaired decidualization. Our findings are supported by reports showing that ablation of COUP-TFII in the uterus results in implantation and placenta formation failure [41, 42] and that deletion or mutation of KIF3B impairs ciliary function and causes retinal ciliopathy [43, 44]. These results demonstrate that COUP-TFII-regulated ESC ciliogenesis is required for decidualization, and inhibition of COUP-TFII by TGF-β1 results in reduced uterine receptivity. This finding also explains why fertility is severely reduced in women with endometriosis.

Endometriosis is one of the leading causes of female infertility. Despite the assistance of reproductive technologies, the pregnancy rate of women with endometriosis remains low [45]. We found that decidual markers are reduced in women with endometriosis compared to their normal counterparts, indicating that uterine receptivity is impaired in women with endometriosis. The concentrations of TGF-β1 are higher in the peritoneal and uterine fluids of women with endometriosis and are negatively associated with the lengths and percentage of primary cilia. Treatment with peritoneal fluid from women with endometriosis inhibits ciliogenesis in ESCs. In conjunction with our previous study that the expression of COUP-TFII in human ESCs is inhibited by TGF-β1 [37], our current data demonstrate that the reduced uterine receptivity in women with endometriosis is mediated, at least in part, by elevated TGF-β1-suppressed COUP-TFII expression and COUP-TFII-dependent ciliogenesis.

Our study demonstrates that TGF-β1 disrupts decidualization, leading to impaired uterine receptivity in women with endometriosis, which is consistent with reports showing that constitutive activation of TGF-β receptor 1 (TGFBR1, also known as ALK5) leads to disruption of the uterine environment required for successful pregnancy [46, 47]. Furthermore, a recent single-cell RNA sequencing study found that TGF-β signaling is gradually inhibited from the proliferative to the late secretory phase, indicating that a controlled reduction in TGF-β signaling may be necessary for proper decidualization and uterine receptivity [48]. Interestingly, conditional *TGFBR1* knockout mice indicate that loss of *TGFBR1* does not impair decidualization or implantation, it leads to post-implantation defects, including restricted fetal growth and impaired placental development [49]. This indicates that in normal pregnancy, TGF-β signaling plays a more crucial role after implantation. Additionally, TGFBR1 is critical for maintaining uterine structural integrity, as *TGFBR1*-deficient mice develop oviductal malformations and impaired embryo transport, contributing to infertility [50]. These findings emphasize the importance of precise spatiotemporal regulation of TGF-β signaling for pregnancy success**.**

The in vitro mechanistic study is supported by in vivo mouse studies and clinical observations. In the mouse model, intra-uterine injection of TGF-β1 decreases the percentage of ciliated cells, reduces the length of cilia, downregulates nuclear p-CREB, inhibits decidualization, and decreases the embryo implantation rate. In contrast, pretreatment with TGF-β receptor inhibitor abolishes the action of TGF-β1, as the ESCs’ decidualization capability is restored and the mice implantation rate is rescued. In women undergoing IVF-ET, embryo implantation, and term birth rates are positively associated with the length of primary cilia and the percentage of ciliated ESCs. Particularly, in a woman who underwent multiple IVF cycles, the success of pregnancy is positively correlated with the endometrial ESCs’ primary cilia number and length. Ciliary length and the percentage of ciliated cells achieved AUCs of 0.86 and 0.79, respectively. These values significantly outperform the AUC of 0.52 reported in a recent ERA clinical trial, which relies on a 238-gene transcriptomic signature to predict the window of implantation [51].

## Conclusions

Primary cilium is indispensable for the decidualization of ESCs, and EP4-transmitted PGE_2_ signaling plays a crucial role. The level of COUP-TFII is downregulated by TGF-β1, resulting in reduced ciliation, shortening of the primary cilium, and decreased decidualization capability, which results in reduced fertility (Fig. 7I). Animal models and human clinical data provide solid evidence to support the notion that the primary cilium is indispensable for maintaining a receptive uterus. Collectively, our study reveals a novel mechanism by which TGF-β1 impairs uterine receptivity through the inhibition of primary cilia in infertile and endometriosis patients. Targeting TGF-β1 or restoring primary cilia-mediated cAMP signaling may have therapeutic potential for treating infertility due to reduced uterine receptivity.

## Supplementary Information

Supplementary Material 1.

## Data Availability

Data for Fig. 4A, S6A, and S6B were derived from the GEO dataset GSE120103. Data for Fig. 4F and Figure S6C were obtained from the GEO dataset GSE107469. Figure S3G was generated by using the UCSC Genome Browser (https://genome.ucsc.edu/).

## Abbreviations

cAMP: Cyclic adenosine monophosphate
COUP-TFII: Chicken ovalbumin upstream promoter transcription factor II
CREB: CAMP response element-binding protein
DMSO: Dimethyl sulfoxide
dpc: Days post coitum
E2: 17β-Estradiol
ELISA: Enzyme-linked immunosorbent assay
EP2/EP4: Prostaglandin E_2_ receptors 2 and 4
EPC: Estradiol + progesterone (MPA) + cAMP cocktail
EPG: Estradiol + progesterone (MPA) + prostaglandin E₂ cocktail
ESCs: Endometrial stromal cells
FOXO1: Forkhead box protein O1
GEO: Gene expression omnibus
Hh: Hedgehog
HOXA10: Homeobox A10
IFT88: Intraflagellar transport 88
IGFBP1: Insulin-like growth factor binding protein 1
IL-6: Interleukin-6
IVF-ET: In vitro fertilization–embryo transfer
KIF3B: Kinesin family member 3B
MPA: Medroxyprogesterone acetate
NP: Non-pregnant
PBS: Phosphate-buffered saline
PGE₂: Prostaglandin E_2_
PKA: Protein kinase A
PGR: Progesterone receptor
PRL: Prolactin
qPCR: Quantitative polymerase chain reaction
ROC: Receiver operating characteristic
SB431542: Selective TGF-β type I receptor inhibitor
TGF-β1: Transforming growth factor beta 1
TNF-α: Tumor Necrosis Factor-α
