# Supplementary material for: Disruption of primary ciliary prostaglandin E2 signaling by transforming growth factor-β1 impairs endometrial receptivity
Source: J Biomed Sci. 2026 Mar 11;33:28. doi: 10.1186/s12929-026-01233-2 (PMC12980961; doi:10.1186/s12929-026-01233-2)
Supplement: Supplementary file 1 — Supplementary Material 1. [file 12929_2026_1233_MOESM1_ESM.docx]

**Supplementary Information**

**Supplementary Methods**

**Clinical samples from patients**

Endometrial tissues or peritoneal fluid from women with or without endometriosis were collected during hysteroscopic surgery or laparoscopic operations at National Cheng Kung University Hospital. Women with endometrial polyps, cervical polyps, leiomyomas (uterine fibroids), fallopian tube obstruction, or dysmenorrhea requiring surgical intervention were included and defined as the control group (without endometriosis). Women with adenomyosis were excluded from this study. For the IVF-ET, the oocytes were retrieved, subjected to IVF, and the embryos were frozen according to standard procedure. One to several cycles later, depending on patient availability due to personal reasons other than disease, endometrial samples were collected via hysteroscopy. Embryo transfer was conducted one or two cycles after the endometrial sample collection. The procedure for peritoneal fluid collection has been described previously [3]. Peritoneal fluid was collected from individual patients in the secretory phase prior to pelvic surgery using a follicular aspiration needle during laparoscopy. The pelvis was inspected to exclude pelvic inflammatory disease. The fluid was collected sterilely, centrifuged at 500 × g for 10 minutes, and the cell-free supernatant was stored at –80 °C. Peritoneal fluids from five normal and five endometriosis patients were pooled, aliquoted, and frozen for subsequent cell treatment assays. Uterine fluid was collected using the uterine cavity irrigation method [1]. Briefly, 2.5 ml sterile saline was gently instilled into the uterine cavity via syringe before hysteroscopic surgery, avoiding contamination from the cervix or vagina. The collected fluid was centrifuged at 1000 × g for 10 minutes to remove cells and stored at –80 °C.

**Primary ESC isolation and treatment**

The procedure for the isolation of ESCs from patients with or without endometriosis was described previously [2]. In brief, tissues were rinsed with PBS and digested with type IV collagenase (2 mg/ml) at 37°C for 1 hour with agitation. Stromal cells were separated from epithelial glands by filtration through 70-μm pore size and then 40-μm pore size nylon meshes. Filtrated cells were allowed to attach in a T-75 flask for approximately 30 min, after which blood cells and debris were washed off by rinsing with PBS. Stromal cells were cultured in DMEM/F12 (Thermo Fisher Scientific) supplemented with 10% fetal bovine serum (FBS, Thermo Fisher Scientific, USA) and 0.2% penicillin-streptomycin in a humidified atmosphere with 5% CO_2_ at 37 °C. For the induction of ciliogenesis and cytokine treatment, ESCs were cultured in a serum-free medium for 48 hours in the presence of recombinant TGF-β1 (PeproTech, 100-21). For the *in vitro* decidualization, ESCs were treated with DMEM/F12 containing 10 nM 17β-estradiol (Sigma-Aldrich, E2758), 1 μM medroxyprogesterone acetate (Sigma-Aldrich, M1629), and 50 μM 8-bromo-cAMP (Sigma-Aldrich, B5368), the EPC medium, under serum-free conditions or with 10% charcoal-stripped fetal bovine serum to induce decidualization for 4-6 days. Half of the culture medium was replaced with EPC medium every 48 hours. For primary cilia manipulation, ESCs were treated with 20 μM roscovitine (Sigma-Aldrich, R7772) for 48 hours. In the PGE_2_-induced decidualization system, ESCs were treated with EPG medium, which is a mixture of 10 nM 17β-estradiol, 1 μM medroxyprogesterone acetate, and 1 μM PGE_2_ (Cayman Chemical, 14010) in DMEM/F12 to induce decidualization for 24 hours or 4 days. For Hedgehog signaling activation, ESCs were treated with 10, 20 ng/ml recombinant human SHH (STEMCELL Technologies, 78065.1), or 200 μM SAG (Selleck, S7779) combined with EPC medium or not for 4 or 6 days. For EP4 activation, ESCs were treated with 10 μM or 1 μM CAY10598 (Cayman Chemical, 13281) for 4 hours or 4 days, respectively.

**Pseudopregnancy and pregnancy mouse models**

Eight-week-old C57BL/6 mice were obtained from the Animal Center at the College of Medicine, National Cheng Kung University. In the pseudopregnancy model, female mice were paired with vasectomized males on day 0 (0 dpc), and the presence of a vaginal plug the following morning was designated as day 0.5 (0.5 dpc). At 0.5 dpc, after anesthetizing the mice with isoflurane, the fur on their backs was shaved, and the skin was disinfected with alcohol. Two small incisions were made, and the mice received intrauterine injections of either TGF-β1 (10 ng in 25 μl PBS per uterine horn) or an equivalent volume of PBS (control). At 4.5 dpc, which corresponds to the typical implantation window in mice, the animals were euthanized, and their uteri were collected for analysis by immunofluorescence and immunohistochemistry staining. In the pregnant mouse model, female mice were paired with fertile males on 0 dpc, with the detection of a vaginal plug occurring at 0.5 dpc. At 1.5 dpc, following a similar procedure to that used in the pseudopregnancy model, the uteri of the female mice were injected with either TGF-β1 (10 ng in 25 μl per uterine horn) or an equal volume of PBS. At 7.5 dpc, the mice were intravenously injected with 1% Evans blue through the tail vein before being sacrificed. To investigate the effects of a TGF-β1 receptor inhibitor on the implantation rate in TGF-β1-treated mice, a protocol similar to the pregnant mouse model was followed, except that either 4 mg/ml SB431542 (25 μl per uterine horn, Cayman Chemicals, 13031) or an equal volume of 20% DMSO (control) was injected 30 minutes before administration of TGF-β1. All animal procedures were approved by the Institutional Animal Care and Use Committee at National Cheng Kung University.

**Lentivirus infection**

For the lentivirus-mediated shRNA knockdown system, the lentiviruses were obtained from the RNA Technology Platform and Gene Manipulation Core Facility (RNAi core) of the National Core Facility for Biopharmaceuticals at Academia Sinica, Taiwan. ESCs were infected with the lentivirus packaged with sh*IFT88* or sh*CEP164* at 37 °C for 48 hours. The viral supernatant was removed, and the cells were then cultured in media containing EPC or CAY10598 for 4-6 days to induce decidualization.

**siRNA and plasmid transfection**

The ESCs were seeded in 6-well plates at a density of 3 × 10^5 cells per well. Cells were transfected with control siRNA (siNC) or COUP-TFII siRNA (siCII, Thermo Fisher s41023) at a final concentration of 40 nM using Lipofectamine 2000 (Thermo Fisher Scientific), according to the manufacturer’s instructions. Twenty-four hours after siRNA transfection, cells were subsequently transfected with either an empty vector or a KIF3B expression plasmid (Sino Biological) using Lipofectamine 2000. For plasmid transfection, 1.5 µg of plasmid DNA was used per well.

**Bioinformatic analysis**

Gene expression profiles from fertile women without endometriosis (n=9) and infertile women with endometriosis (n=9) were obtained from the Gene Expression Omnibus (GEO) database, specifically from microarray dataset GSE120103. Differentially expressed genes in infertile women with endometriosis, defined by a log2 fold change < -2 and FDR < 0.01, were identified and compared with a set of 426 genes associated with decidualization. For the analysis of gene expression in *COUP-TFII*-knockdown ESCs, raw data from the microarray dataset GSE107469 were processed. Genes with an FDR < 0.01 were selected and overlapped with genes annotated in the following Gene Ontology categories: GO:0031513, GO:0060271, GO:0097730, and GO:1902855. To predict p-CREB binding sites on the *IGFBP1* promoter region, ChIP-seq datasets for p-CREB available in ENCODE were visualized using the UCSC Genome Browser. The potential p-CREB binding motifs near the *IGFBP1* promoter region were identified using the JASPAR database.

**Table S1: Baseline characteristics of women who underwent IVF-ET**

|  | **Pregnant (n=29)**  **mean ± SD** | **Non-pregnant (n=22)**  **mean ± SD** | **P value** |
| --- | --- | --- | --- |
| **BMI** | $24.4\pm4.3$ | $23.4\pm3.2$ | 0.376 |
| **Age** | $37\pm4.7$ | $39\pm6.3$ | 0.221 |
| **Gravidity** | $0.8\pm1.7$ | $0.9\pm1.0$ | 0.826 |
| **Parity** | $0.2\pm0.5$ | $0.2\pm0.4$ | 0.901 |
| **AMH (ng/ml)** | $2.7\pm2.0$ | $1.6\pm0.9$ | 0.018 |
| **Endometrium thickness (mm),** | $11.1\pm2.0$ | $10.3\pm2.1$ | 0.146 |
| **Type of infertility** |  |  |  |
| Primary | 72.4% (21/29) | 72.7% (16/22) |  |
| Secondary | 20.7% (6/29) | 22.7% (5/22) |  |
| Non-infertility indication (e.g., PGD) | 6.9% (1/29) | 4.5% (1/22) |  |
| **Type of transferred embryo** |  |  |  |
| Fresh | 3.4% (1/29) | 0% (0/22) |  |
| Frozen | 96.6% (28/29) | 100% (22/22) |  |
| **No. of transferredembryoso** |  |  |  |
| 1 | 31.0% (9/29) | 50% (11/22) |  |
| 2 | 62.1% (18/29) | 45.5% (10/22) |  |
| 3 | 6.9% (2/29) | 4.5% (1/22) |  |
| **Embryo stage** |  |  |  |
| Cleavage | 24.1% (7/29) | 36.4% (8/22) |  |
| Blastomere | 69.0% (20/29) | 45.5% (10/22) |  |
| Early blastocyst | 6.9% (2/29) | 18.2% (4/22) |  |
| **Live Birth rate** | 82.8% (24/29) | Non-applicable |  |

**Table S2: IVF-ET women characteristics and embryo information**

| IVF outcome: pregnant | Age | BMI | Embryo stage | Embryos Transferred | Embryo grade | Phase |
| --- | --- | --- | --- | --- | --- | --- |
| #1 | 37 | 27.7 | Blastomere | 2 | 5BC, 2BC | S |
| #2 | 40 | 23.1 | Cleavage stage | 3 | 1,1,4 | S |
| #3 | 33 | 20.9 | Early blastocyst | 1 | EB | S |
| #4 | 34 | 28.3 | Blastomere | 2 | 5BA, 5BA | S |
| #5 | 39 | 19.3 | Blastomere | 1 | 4BB | S |
| #6 | 34 | 18.8 | Cleavage stage | 2 | 1, 3 | S |
| #7 | 36 | 29.1 | Early blastocyst | 1 | EB | S |
| #8 | 41 | 19.6 | Blastomere | 2 | 6AA, 5BB | S |
| #9 | 31 | 30.4 | Cleavage stage | 2 | 2,4 | S |
| #10 | 37 | 23.2 | Cleavage stage | 3 | 1,2,3 | S |
| #11 | 32 | 21.7 | Blastomere | 1 | 5BC | S |
| #12 | 44 | 26.2 | Cleavage stage | 2 | 2, 2 | S |
| #13 | 40 | 27.5 | Cleavage stage | 2 | 3, 3 | S |
| #14 | 33 | 26 | Blastomere | 2 | 6AA, 5AB | S |
| #15 | 37 | 20.9 | Blastomere | 2 | 4AB, 4BC | S |
| #16 | 46 | 31.1 | Cleavage stage | 2 | 1, 2 | S |
| #17 | 38 | 23.2 | Blastomere | 2 | 5BB, 6AA | S |
| #18 | 38 | 27.8 | Blastomere | 2 | 5BB, 5BC | S |
| #19 | 34 | 22 | Blastomere | 2 | 6BA, 5BB | S |
| #20 | 40 | 22.7 | Blastomere | 2 | 5BA, 5BA | S |
| #21 | 27 | 19.9 | Blastomere/Early blastocyst | 2 | 5BC, EB | S |
| #22 | 40 | 26.3 | Blastomere | 2 | 5BB, 5BB | S |
| #23 | 32 | 25 | Blastomere | 1 | 5BD | S |
| #24 | 39 | 21.6 | Blastomere | 2 | 5BB, 3BB | S |
| #25 | 40 | 32 | Blastomere | 2 | 5BC, 5BC | S |
| #26 | 39 | 32 | Blastomere | 2 | 4BB, 2BB | S |
| #27 | 43 | 18 | Blastomere | 1 | 5BC | S |
| #28 | 27 | 24.2 | Blastomere | 1 | 5BB | S |
| #29 | 42 | 18.2 | Blastomere | 1 | 5BA | S |
| IVF outcome: non-pregnant | **Age** | **BMI** | **Embryo stage** | **Embryos Transferred** | **Embryo grade** | **Phase** |
| #1 | 28 | 25.6 | Early blastocyst | 1 | EB | S |
| #2 | 29 | 26 | Blastomere | 1 | 5BB | S |
| #3 | 37 | 21 | Blastomere | 2 | 6AA, 5BB | S |
| #4 | 38 | 20.8 | Blastomere | 2 | 5AA, 5BB | S |
| #5 | 44 | 27.8 | Cleavage stage | 2 | 3, 3 | S |
| #6 | 45 | 26.1 | Early blastocyst | 1 | EB | S |
| #7 | 40 | 20.9 | Blastomere | 2 | 5AB, 5BC | S |
| #8 | 45 | 29.6 | Early blastocyst | 2 | EB, EB | S |
| #9 | 46 | 29.2 | Blastomere/Morula | 3 | 5BB, 5BC, Morula | S |
| #10 | 29 | 24 | Early blastocyst | 1 | EB | S |
| #11 | 29 | 22.5 | Cleavage stage | 1 | 3 | S |
| #12 | 40 | 22.7 | Cleavage stage | 1 | 1 | S |
| #13 | 40 | 21.8 | Blastomere | 1 | 4BC | S |
| #14 | 41 | 23.9 | Blastomere | 2 | 5BB, 5AC | S |
| #15 | 47 | 20 | Cleavage stage | 2 | 2, 2 | S |
| #16 | 45 | 23.2 | Cleavage stage | 2 | 3, 3 | S |
| #17 | 49 | 18.3 | Cleavage stage | 1 | 1 | S |
| #18 | 39 | 21.2 | Blastomere | 2 | 5BB, 5BB | S |
| #19 | 38 | 26.3 | Early blastocyst | 1 | EB | S |
| #20 | 34 | 22.8 | Blastomere | 1 | 6BD | S |
| #21 | 33 | 22 | Blastomere | 1 | 6BB | S |
| #22 | 40 | 18.4 | Cleavage stage | 2 | 2,4 | S |

EB: early blastocyst; S: secretory phase

**Table S3. Patient information and experimental application**

| # | Normal or Endometriosis | Description | Phase | Age | Cell culture | IHC/ IF staining | ELISA assay |
| --- | --- | --- | --- | --- | --- | --- | --- |
| N1 | Normal | Leiomyoma | S | 47 | + | - | - |
| N2 | Normal | Uterine myoma | S | 36 | + | - | - |
| N3 | Normal | Uterine myoma | S | 44 | + | - | - |
| N4 | Normal | Hydrosalpinx | S | 33 | + | - | - |
| N5 | Normal | Endometrial polyp | S | 40 | + | - | - |
| N6 | Normal | Leiomyoma | P | 37 | + | - | - |
| N7 | Normal | Pelvic adhesion | P | 31 | + | - | - |
| N8 | Normal | Leiomyoma | S | 35 | + | - | - |
| N9 | Normal | Pelvic adhesion | S | 28 | + | - | - |
| N10 | Normal | Leiomyoma | P | 45 | + | - | - |
| N11 | Normal | Pelvic adhesion | P | 37 | + | - | - |
| N12 | Normal | Uterine myoma | S | 46 | + | - | - |
| N13 | Normal | Endometrial polyp | P | 32 | + | + | - |
| N14 | Normal | Endometrial polyp | P | 44 | - | + | + |
| N15 | Normal | Endometrial polyp | P | 43 | - | + | + |
| N16 | Normal | Bilateral tubal obstruction | P | 44 | - | + | + |
| N17 | Normal | Endometrial polyp | P | 43 | - | + | - |
| N18 | Normal | Leiomyoma | P | 45 | - | + | - |
| N19 | Normal | Uterine myoma | P | 37 | - | + | - |
| N20 | Normal | Uterine myoma | P | 47 | - | + | - |
| N21 | Normal | Endometrial polyp | P | 32 | - | + | - |
| N22 | Normal | Endometrial polyp | P | 41 | - | + | - |
| N23 | Normal | Leiomyoma | P | 37 | - | + | - |
| N24 | Normal | Cervical polyp | S | 42 | - | + | - |
| N25 | Normal | Fallopian tube obstruction | S | 39 | - | + | + |
| N26 | Normal | Endometrial polyp | S | 40 | - | + | + |
| N27 | Normal | Uterine myoma | S | 46 | - | + | - |
| N28 | Normal | Endometrial polyp | S | 32 | - | + | + |
| N29 | Normal | Endometrial polyp | S | 38 | - | + | + |
| N30 | Normal | Mild cervical dysplasia | S | 38 | - | + | + |
| N31 | Normal | Leiomyoma | S | 41 | - | + | + |
| N32 | Normal | Endometrial polyp | S | 39 | - | + | - |
| N33 | Normal | Fallopian tube obstruction | S | 31 | - | + | - |
| N34 | Normal | Mild cervical dysplasia | S | 46 | - | + | - |
| N35 | Normal | Fallopian tube obstruction | S | 32 | - | + | - |
| N36 | Normal | Polycystic ovary syndrome | S | 38 | - | + | - |
| N37 | Normal | Cervical polyp | S | 42 | - | + | - |
| N38 | Normal | Pelvic congestion syndrome | S | 38 | - | + | - |
| N39 | Normal | Fallopian tube obstruction | S | 44 | - | + | - |
| N40 | Normal | Uterine myoma | S | 47 | - | + | - |
| N41 | Normal | Endometrial polyp | S | 38 | - | + | - |
| N42 | Normal | Mild cervical dysplasia | S | 34 | - | + | + |
| N43 | Normal | Endometrial polyp | S | 41 | - | + | + |
| N44 | Normal | Leiomyoma | S | 33 | - | + | + |
| N45 | Normal | Endometrial polyp | S | 41 | - | + | + |
| N46 | Normal | Endometrial polyp | S | 39 | - | + | + |
| N47 | Normal | Leiomyoma | S | 44 | - | + | + |
| N48 | Normal | Fallopian tube obstruction | S | 47 | - | + | + |
| N49 | Normal | Leiomyoma | S | 39 | - | + | + |
| N50 | Normal | Fallopian tube obstruction | S | 38 | - | + | + |
| N51 | Normal | Fallopian tube obstruction | P | 29 | - | - | + |
| N52 | Normal | Fallopian tube obstruction | P | 38 | - | - | + |
| N53 | Normal | Leiomyoma | P | 38 | - | - | + |
| N54 | Normal | Leiomyoma | P | 36 | - | - | + |
| N55 | Normal | Leiomyoma | S | 31 | - | - | + |
| N56 | Normal | Fallopian tube obstruction | S | 32 | - | - | + |
| N57 | Normal | Leiomyoma | P | 32 | - | - | + |
| N58 | Normal | Uterine retroversion | S | 34 | - | - | + |
| N59 | Normal | Fallopian tube obstruction | S | 30 | - | - | + |
| N60 | Normal | Fallopian tube obstruction | P | 39 | - | + | - |
| N61 | Normal | Fallopian tube obstruction | P | 37 | - | + | - |
| N62 | Normal | Leiomyoma | P | 44 | - | + | + |
| N63 | Normal | Dysmenorrhea | S | 48 | - | + | + |
| N64 | Normal | Uterine myoma | P | 35 | - | + | - |
| N65 | Normal | Leiomyoma | P | 40 | - | + | - |
| N66 | Normal | Pelvic peritoneal adhesions | S | 28 | - | + | + |
| N67 | Normal | Mucinous cystadenoma | P | 45 | - | + | - |
| N68 | Normal | Leiomyoma | S | 40 | - | + | + |
| N69 | Normal | Leiomyoma | S | 36 | - | + | + |
| N70 | Normal | Leiomyoma | S | 43 | - | + | + |
| N71 | Normal | Endometrial polyp | S | 40 | - | - | + |
| N72 | Normal | Endometrial polyp | S | 46 | - | - | + |
| N73 | Normal | Endometrial polyp | S | 38 | - | - | + |
| N74 | Normal | Endometrial polyp | P | 30 | - | + | - |
| N75 | Normal | Dysmenorrhea | S | 33 | - | - | + |
| N76 | Normal | Endometrial polyp | P | 34 | - | + | - |
| N77 | Normal | Hyperprolactinemia | S | 37 | - | - | + |
| N78 | Normal | Leiomyoma | S | 44 | - | - | + |
| N79 | Normal | Leiomyoma | P | 47 | - | + | - |
| E1 | Endometriosis | ASRM II | P | 43 | - | + | - |
| E2 | Endometriosis | ASRM IV | S | 39 | + | + | - |
| E3 | Endometriosis | ASRM III | P | 38 | - | + | - |
| E4 | Endometriosis | ASRM I | S | 37 | - | + | + |
| E5 | Endometriosis | ASRM II | S | 35 | - | + | + |
| E6 | Endometriosis | ASRM IV | S | 43 | - | + | + |
| E7 | Endometriosis | ASRM II | S | 38 | - | + | + |
| E8 | Endometriosis | ASRM II | P | 32 | - | + | - |
| E9 | Endometriosis | ASRM I | S | 36 | - | + | + |
| E10 | Endometriosis | ASRM III | S | 23 | + | + | + |
| E11 | Endometriosis | ASRM III | S | 30 | - | + | + |
| E12 | Endometriosis | ASRM IV | S | 32 | - | + | + |
| E13 | Endometriosis | ASRM IV | S | 22 | - | + | + |
| E14 | Endometriosis | ASRM III | S | 26 | - | + | + |
| E15 | Endometriosis | ASRM I | P | 28 | + | + | - |
| E16 | Endometriosis | ASRMIV | S | 29 | - | + | + |
| E17 | Endometriosis | ASRM III | S | 23 | - | + | - |
| E18 | Endometriosis | ASRM III | S | 23 | - | + | - |
| E19 | Endometriosis | ASRM IV | S | 35 | - | + | + |
| E20 | Endometriosis | ASRM II | S | 39 | - | + | + |
| E21 | Endometriosis | ASRM IV | S | 33 | - | - | + |
| E22 | Endometriosis | ASRM IV | S | 35 | - | - | + |
| E23 | Endometriosis | ASRM II | S | 48 | - | + | + |
| E24 | Endometriosis | ASRM III | S | 33 | - | - | + |
| E25 | Endometriosis | ASRM III | P | 24 | - | + | - |
| E26 | Endometriosis | ASRM II | S | 38 | - | - | + |
| E27 | Endometriosis | ASRM III | S | 36 | - | - | + |
| E28 | Endometriosis | ASRM III | S | 36 | - | - | + |
| E29 | Endometriosis | ASRMII | S | 37 | - | + | + |
| E30 | Endometriosis | ASRM IV | P | 34 | + | + | - |
| E31 | Endometriosis | ASRM II | S | 37 | - | - | + |
| E32 | Endometriosis | ASRM IV | S | 40 | - | + | + |
| E33 | Endometriosis | ASRM IV | S | 37 | - | - | + |
| E34 | Endometriosis | ASRM IV | P | 37 | - | + | - |
| E35 | Endometriosis | ASRM IV | P | 42 | - | + | - |
| E36 | Endometriosis | ASRM IV | S | 38 | - | + | + |
| E37 | Endometriosis | ASRM IV | P | 24 | - | + | - |
| E38 | Endometriosis | ASRM IV | P | 40 | - | + | - |
| E39 | Endometriosis | ASRM II | S | 44 | - | - | + |
| E40 | Endometriosis | ASRM IV | S | 45 | - | + | - |
| E41 | Endometriosis | ASRM II | S | 43 | - | + | + |
| E42 | Endometriosis | ASRM IV | S | 45 | - | + | + |
| E43 | Endometriosis | ASRM I | S | 37 | - | - | + |
| E44 | Endometriosis | Unspecified (w/o laparoscopy) | S | 36 | - | - | + |
| E45 | Endometriosis | ASRM IV | P | 39 | - | + | - |
| E46 | Endometriosis | Unspecified (w/o laparoscopy) | S | 47 | - | - | + |
| E47 | Endometriosis | ASRM I | S | 37 | - | + | + |
| E48 | Endometriosis | ASRM I | S | 30 | - | + | + |
| E49 | Endometriosis | ASRM IV | S | 35 | - | - | + |
| E50 | Endometriosis | ASRM III | S | 30 | - | + | + |
| E51 | Endometriosis | ASRM IV | S | 43 | - | - | + |
| E52 | Endometriosis | ASRM IV | S | 29 | - | + | + |
| E53 | Endometriosis | ASRM III | S | 19 | - | + | + |
| E54 | Endometriosis | ASRM IV | S | 38 | - | + | + |
| E55 | Endometriosis | ASRM IV | S | 35 | - | - | + |
| E56 | Endometriosis | ASRM IV | S | 35 | - | - | + |
| E57 | Endometriosis | ASRM IV | S | 34 | - | - | + |
| E58 | Endometriosis | ASRM III | S | 41 | - | - | + |
| E59 | Endometriosis | ASRM IV | S | 31 | - | - | + |
| E60 | Endometriosis | Unspecified (w/o laparoscopy) | S | 36 | - | - | + |
| E61 | Endometriosis | ASRM III | S | 34 | - | - | + |
| E62 | Endometriosis | ASRM III | S | 42 | - | - | + |
| E63 | Endometriosis | Unspecified (w/o laparoscopy) | S | 41 | - | + | + |
| E64 | Endometriosis | Unspecified (w/o laparoscopy) | S | 40 | - | - | + |
| E65 | Endometriosis | Unspecified (w/o laparoscopy) | S | 40 | - | - | + |
| E67 | Endometriosis | Unspecified (w/o laparoscopy) | S | 42 | - | - | + |

*IF: immunofluorescence; IHC: immunohistochemistry; S: secretory phase; P: proliferative phase

**Table S4. Sequence of primers used in this study**

| Primer | Target | sequence (5'-3') |
| --- | --- | --- |
| PRL_realtime_F’ | *Prolactin (PRL)* | GCAGATGGCTGATGAAG |
| PRL_realtime_R’ | *Prolactin (PRL)* | AGCAGTTGTTGTTGTGGATG |
| IGFBP1_realtime_F’ | *IGFBP1* | GGTAGACGCACCAGCAGAG |
| IGFBP1_realtime_R’ | *IGFBP1* | AAGGCACAGGAGACATCAGG |
| FOXO1_realtime_F’ | *FOXO1* | CTACGAGTGGATGGTCAAGAG |
| FOXO1_realtime_R’ | *FOXO1* | ATGAACTTGCTGTGTAGGGAC |
| hGLI1_realtime_F’ | *GLI1* | CATCCTCCAGAACGGCAAGA |
| hGLI1_realtime_R’ | *GLI1* | ACTTTTTCTCTCTCCAGCCGC |
| hPTCH1_realtime_F’ | *PTCH1* | GCGGGATCTGAGTTCGACTTCATT |
| hPTCH1_realtime_R’ | *PTCH1* | GGAAGCAAAACCAGCCCATTGAGA |

**Table S5. Antibody information**

| Antibody | Company | Cat. # | Host species | Application (dilution factor) |
| --- | --- | --- | --- | --- |
| Anti-ARL13B | Proteintech | 17711-1-AP | Rabbit | IF (1:500) |
| Anti-Acetyl-tubulin | Sigma-Aldrich | T7451 | Mouse | IF (1:500) |
| Anti-Progesterone Receptor A/B | Cell signaling | 8757 | Rabbit | IHC (1:500) |
| Anti-FOXO1 | Cell signaling | 2880 | Rabbit | IHC (1:500), IF (1:500), and WB (1:1000) |
| Anti-Ki67 | GeneTex | GTX16667 | Rabbit | IHC (1:250) |
| Anti-COUP-TFII | R&D Systems | PP-H7147-00 | Mouse | IHC (1:500) |
| Anti-COUP-TFII | Cell signaling | 6434 | Rabbit | WB (1:1000) |
| Anti-PKA C alpha / beta | R&D Systems | MAB5908 | Mouse | IF (1:250) |
| Anti-Phospho-CREB | Cell signaling | 9198 | Rabbit | IHC (1:250), IF (1:250), and WB (1:1000) |
| Anti-CREB1 | Santa Cruz | sc-271 | Mouse | IF (1:500) |
| Anti-CREB1 | Santa Cruz | sc-186 | Rabbit | WB (1:1000) |
| Anti-Phospho-SMAD3 (Ser423/425) | Cell signaling | 9520 | Rabbit | IF (1:250) |
| Anti-EP4 | Santa Cruz | sc-55596 | Mouse | IF (1:500) and WB (1:500) |
| Anti-SMO | Santa Cruz | sc-166685 | Mouse | IF (1:500) |
| Anti-SHH | Santa Cruz | sc-9024 | Rabbit | IHC (1:250) and WB (1:500) |
| Macrophage marker | Santa Cruz | sc-66204 | Mouse | IF (1:500) |
| Anti-TGF-β1 | Invitrogen | MA5-15065 | Rabbit | IF (1:200) |
| Hoechst | Invitrogen | H3569 |  | IF (1:10000) |
| Anti-IGFBP1 | Cell signaling | 31025 | Rabbit | WB (1:1000) |
| Anti-KIF3B | Santa Cruz | sc-514165 | Mouse | WB (1:500) |
| Anti-IFT88 | Proteintech | 13967-1-AP | Rabbit | WB (1:1000) |
| Anti-CEP164 | Novus Biologicals | NBP1-81445 | Rabbit | WB (1:1000) |
| Anti-alpha-tubulin | GeneTex | GTX112141 | Rabbit | WB (1:5000) |
| Anti-beta-actin | GeneTex | GTX629630 | Mouse | WB (1:5000) |

IF: immunofluorescence; IHC: immunohistochemistry; WB: Western blotting

**Supplementary Figures**

**
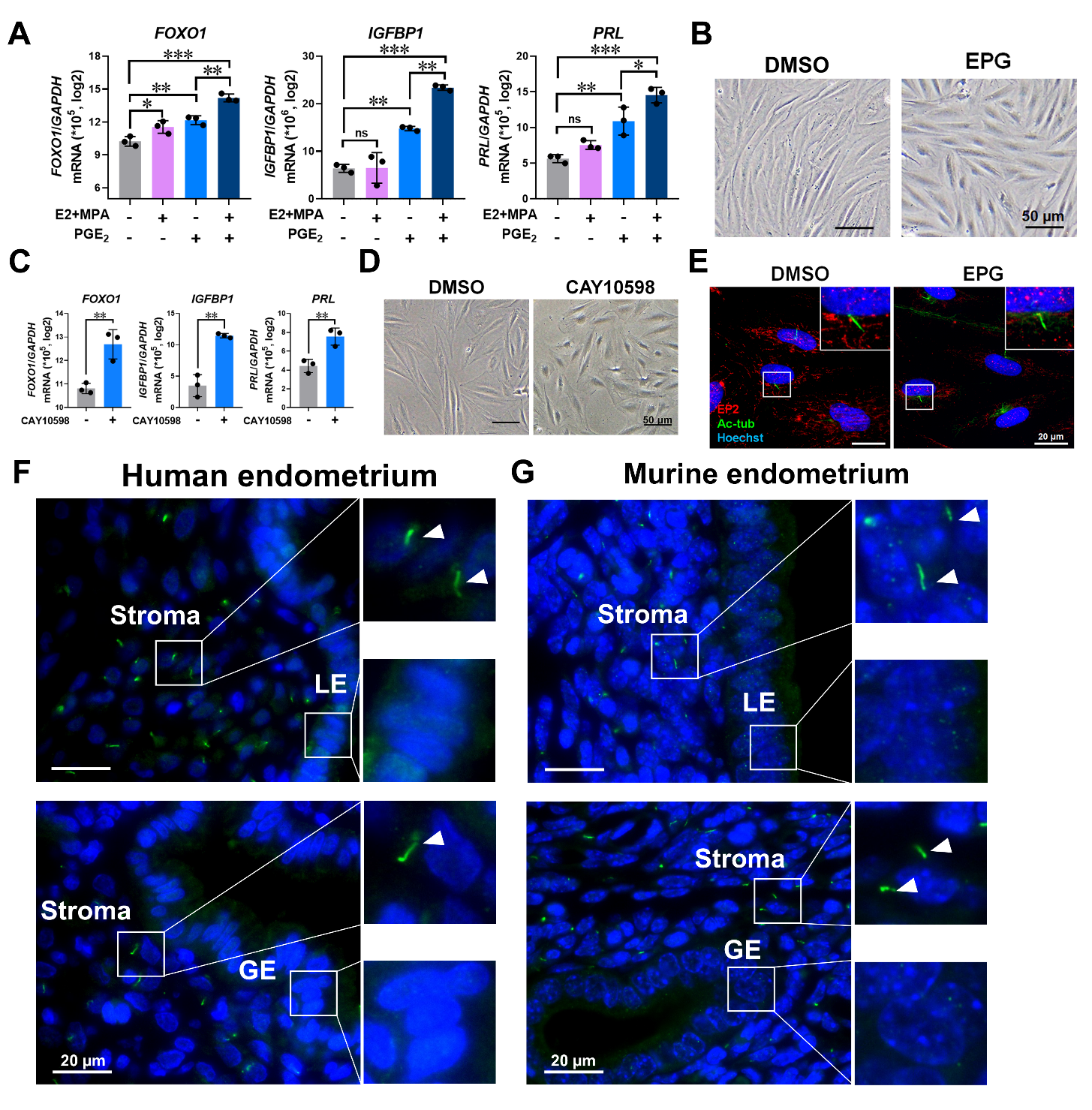
**

**Fig. S1.** **Endometrial stromal cells (ESCs) respond to EPG-induced decidualization and possess primary cilia.** **(A)** The *FOXO1*, *IGFBP1, and PRL* mRNA levels in ESCs after treatment with PGE_2_ (1 μM) and/or medroxyprogesterone acetate (MPA, 1 μM) combined with 17β-estradiol (E_2_, 10 nM) for 4 days (n=4 biological replicates). **(B)** Bright-field images showing ESC morphology after treatment with EPG for 6 days. Scale bar = 50 μm. **(C)** *FOXO1*, *IGFBP1, and PRL* mRNA levels in ESCs after treatment with CAY10598 (1 μM) for 4 days. **(D)** Bright-field images of ESCs treated with DMSO or CAY10598 (1 μM) for 4 days. **(E)** Immunofluorescence staining showing the localization of EP2 (red) and primary cilia (Ac-tubulin, green) in ESCs. Nuclei were stained with Hoechst (blue). Scale bar = 20 μm. **(F and G)** Immunofluorescence staining images of the endometria in humans (F, secretory phase) and mice (G, 4.5 days post-coitum). The anti-ARL13B antibody was used to stain primary cilia (green), and nuclei were stained with Hoechst (blue). Scale bar = 20 μm. Insets show enlarged images of the areas circled in the squares. Arrowheads indicate the primary cilia. LE: luminal epithelium, GE: glandular epithelium. *p ≤ 0.05, **p ≤ 0.01, ***p ≤ 0.001. Statistical significance was determined by Student’s t-test (two groups) or one-way ANOVA with Tukey’s post hoc test (≥3 groups).

**
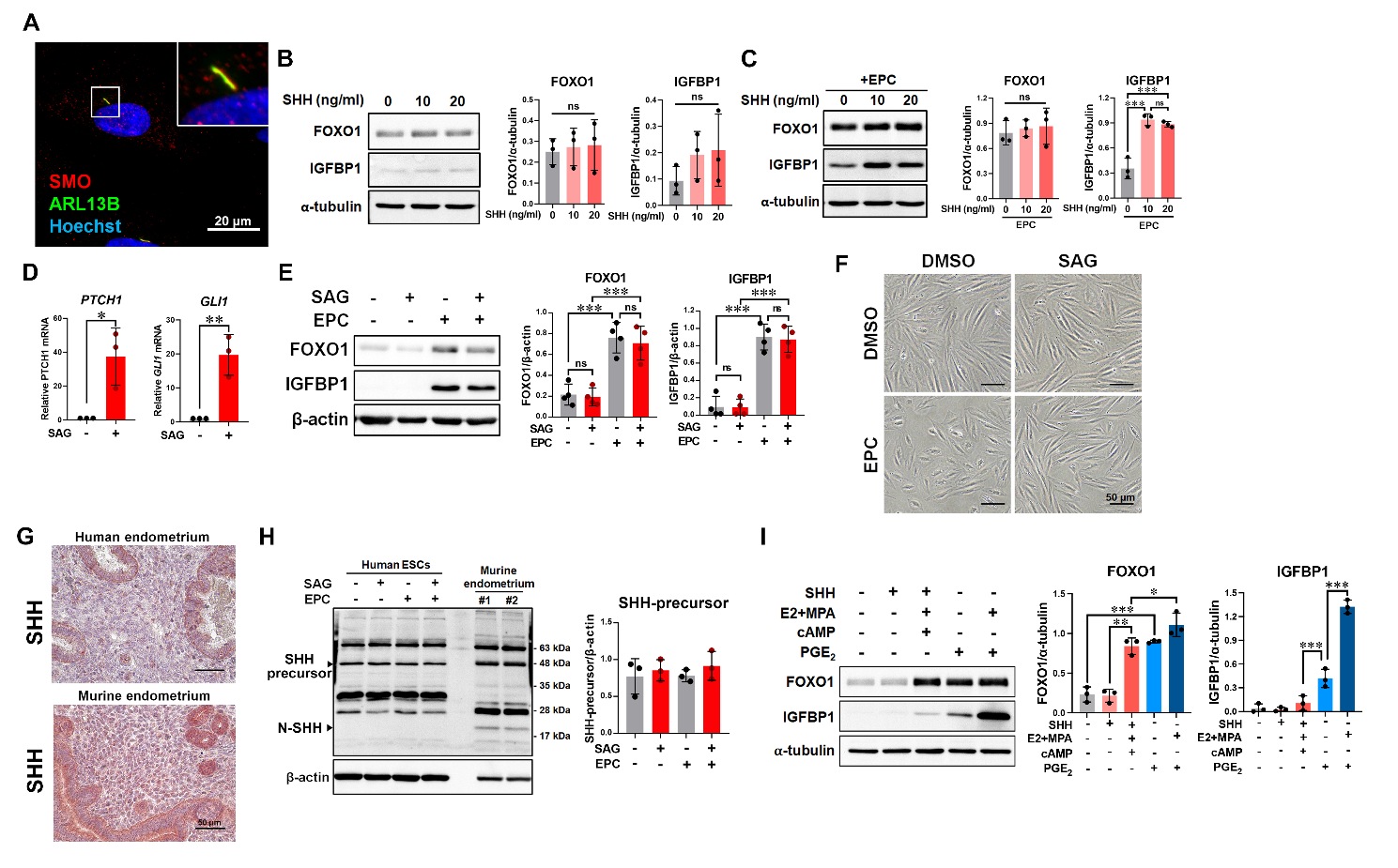
**

**Fig. S2.** **Hedgehog signaling does not involve in the decidualization of human ESCs.** **(A)** Immunofluorescence staining shows the localization of Smoothened (SMO, red) at the primary cilium (anti-ARL13B, green) under serum-free conditions. Nuclei were stained with Hoechst (blue). Scale bar = 20 μm. **(B and C)** Representative Western blot images and quantified results of FOXO1 and IGFBP1 in ESCs treated with recombinant human SHH (10 or 20 ng/ml) in the absence (n=3 biological replicates, B) and presence of EPC (n=3 biological replicates, C) for 4 days. **(D)** mRNA expression of *PTCH1* and *GLI1* after treatment with SAG (200 μM) or DMSO (vehicle) for 6 days (n=3 biological replicates). **(E)** Representative Western blot images and quantified results of FOXO1 and IGFBP1 in ESCs after EPC treatment with or without SAG (200 μM) for 6 days (n=4 biological replicates). **(F)** Bright-field images of ESCs after EPC treatment with or without SAG (200 μM) for 6 days. Scale bar = 50 μm. **(G)** Representative immunohistochemistry staining of SHH in human secretory phase endometrium and murine endometrium derived from 4.5 days post-coitum. Scale bar = 50 μm. **(H)** Representative Western blot images and quantified results of SHH in ESCs treated with SAG/EPC for 6 days (n=3 biological replicates) and in murine endometria from 2 mice. **(I)** Representative Western blot images and quantification of FOXO1 and IGFBP1 protein levels in ESCs treated with SHH (10 ng/ml), 17β-estradiol (E_2_, 10 nM), MPA (1 μM), cAMP (50 μM), and PGE₂ (1 μM) as indicated for 4 days (n=3 biological replicates). Statistical significance was determined by Student’s t-test (two groups) or one-way ANOVA with Tukey’s post hoc test (≥3 groups).


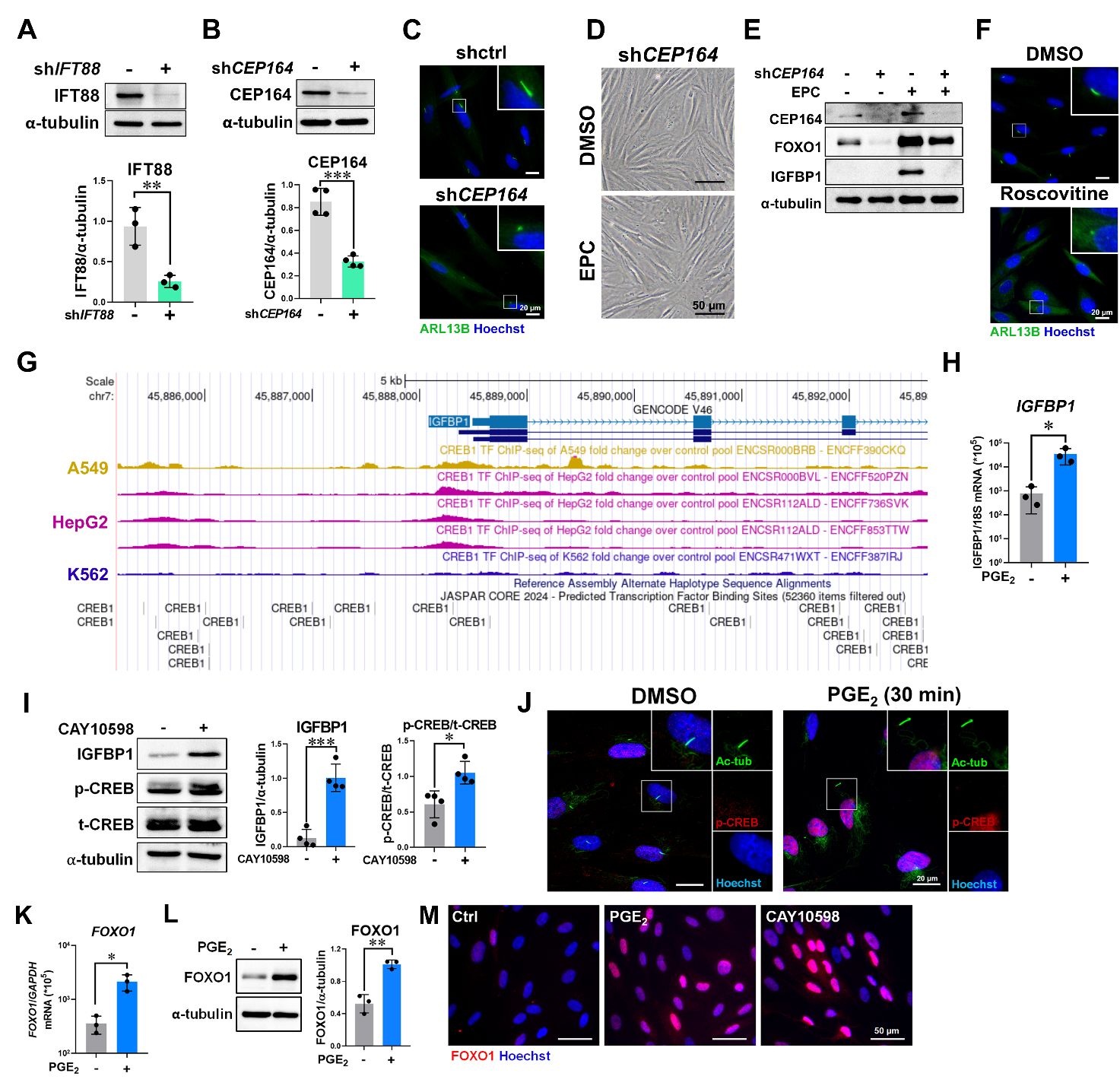


**Fig. S3. PGE_2_ facilitates ciliogenesis and decidualization in human ESCs. (A)** Representative Western blot images and quantified results of IFT88 in *IFT88*-knockdown (sh*IFT88*) and shctrl ESCs (n=3 biological replicates). **(B)** Representative Western blot images and quantified results of CEP164 in *CEP164*-knockdown (sh*CEP164*) and shctrl ESCs (n=4 biological replicates). **(C)** Immunofluorescence images of *CEP164*-knockdown (sh*CEP164*) and control (shctrl) ESCs. Scale bar = 20 μm. **(D)** Bright-field images of shctrl and sh*CEP164* ESCs treated with EPC or DMSO for 6 days. Scale bar = 50 μm. **(E)** Western blot images of CEP164 and decidualization markers post-EPC or DMSO treatment for 6 days. **(F)** Representative immunofluorescence images of ciliogenesis (anti-ARL13B, green) in ESCs treated with roscovitine (20 μM) or DMSO for 48 hours. Scale bar = 20 μm. **(G)** Image extracted from UCSC Genome Browser showing the predicted CREB1 binding sites by JASPAR and ChIP-Seq analysis at the *IGFBP1* promoter region. ChIP-seq results for A549 (lung carcinoma), HepG2 (liver carcinoma), and K562 (chronic myelogenous leukemia) cell lines, provided by ENCODE, identify potential binding sites of CREB1 at the IGFBP1 promoter. **(H)** The *IGFBP1* mRNA levels in ESCs treated with 10 μM PGE_2_ for 4 hours (n=3 biological replicates). **(I)** Representative Western blot images and quantified results of IGFBP1, p-CREB, and t-CREB expression in ESCs treated with 10 μM CAY10598 for 4 hours (n=4 biological replicates). **(J)** Immunofluorescence images showing the localization of p-CREB (red) and primary cilia (Ac-tubulin, green) in ESCs treated with PGE_2_ (10 μM) or DMSO for 30 minutes. Scale bar = 20 μm. **(K and L)** The *FOXO1* mRNA (K) and protein (L) expression in ESCs treated with 10 μM PGE_2_ for 4 hours (n=3 biological replicates). **(M)** Representative immunostaining images of FOXO1 (red) in the nuclei (blue) of ESCs treated with 10 μM PGE_2_ or 10 μM CAY10598 for 4 hours. Nuclei were stained with Hoechst (blue). Scale bar = 50 μm. *p ≤ 0.05, **p ≤ 0.01, ***p ≤ 0.001. Statistical significance was determined by Student’s t-test (two groups) or one-way ANOVA with Tukey’s post hoc test (≥3 groups).


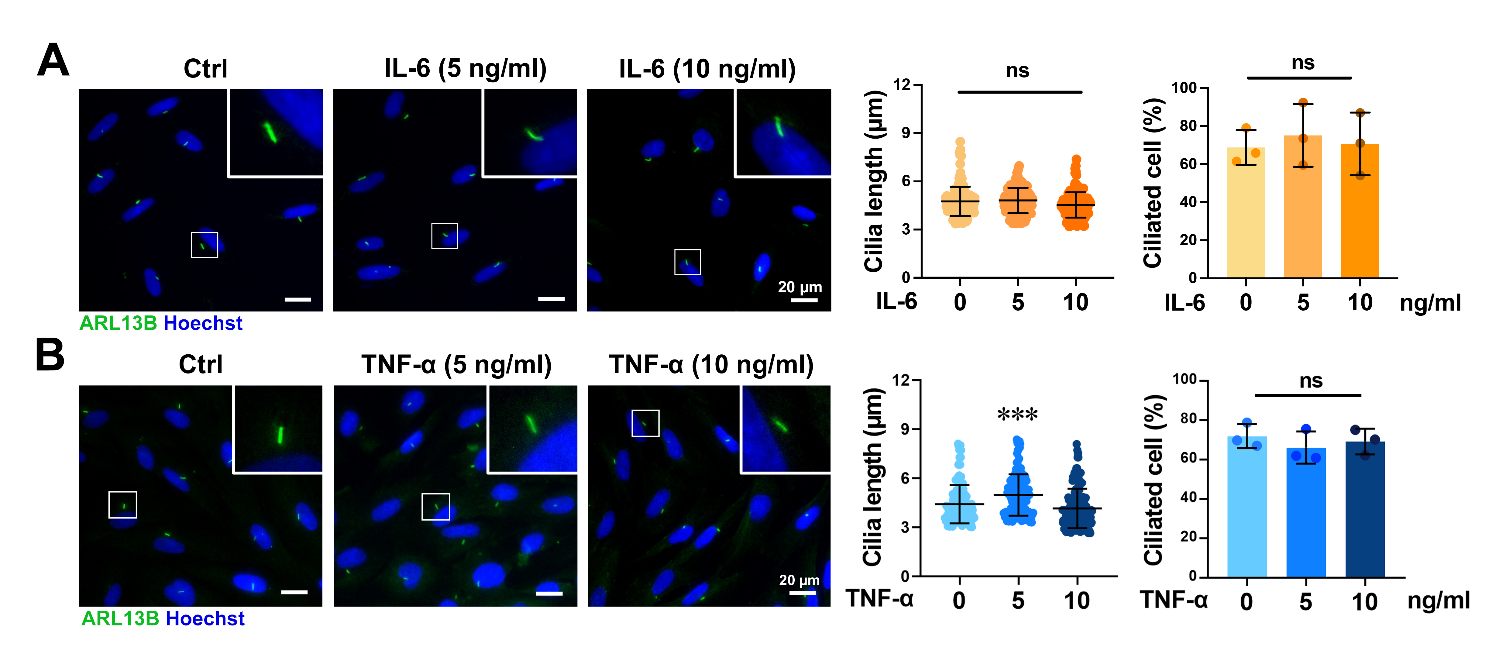


**Fig. S4. The effects of IL-6 and TNF-α on ciliogenesis of ESCs. (A and B)** Representative images and quantitative results of ESC primary cilia (ARL13B, green) after 5 or 10 ng/ml IL-6 (A, n=3 biological replicates), or TNF-α (B, n=3 biological replicates) treatment for 48 hours under serum-free conditions. Cells treated with PBS for 48 hours served as controls (Ctrl). Scale bar = 20 μm. **p ≤ 0.01, ***p ≤ 0.001 by one-way ANOVA followed by Tukey’s multiple comparison test. ns: not significant.


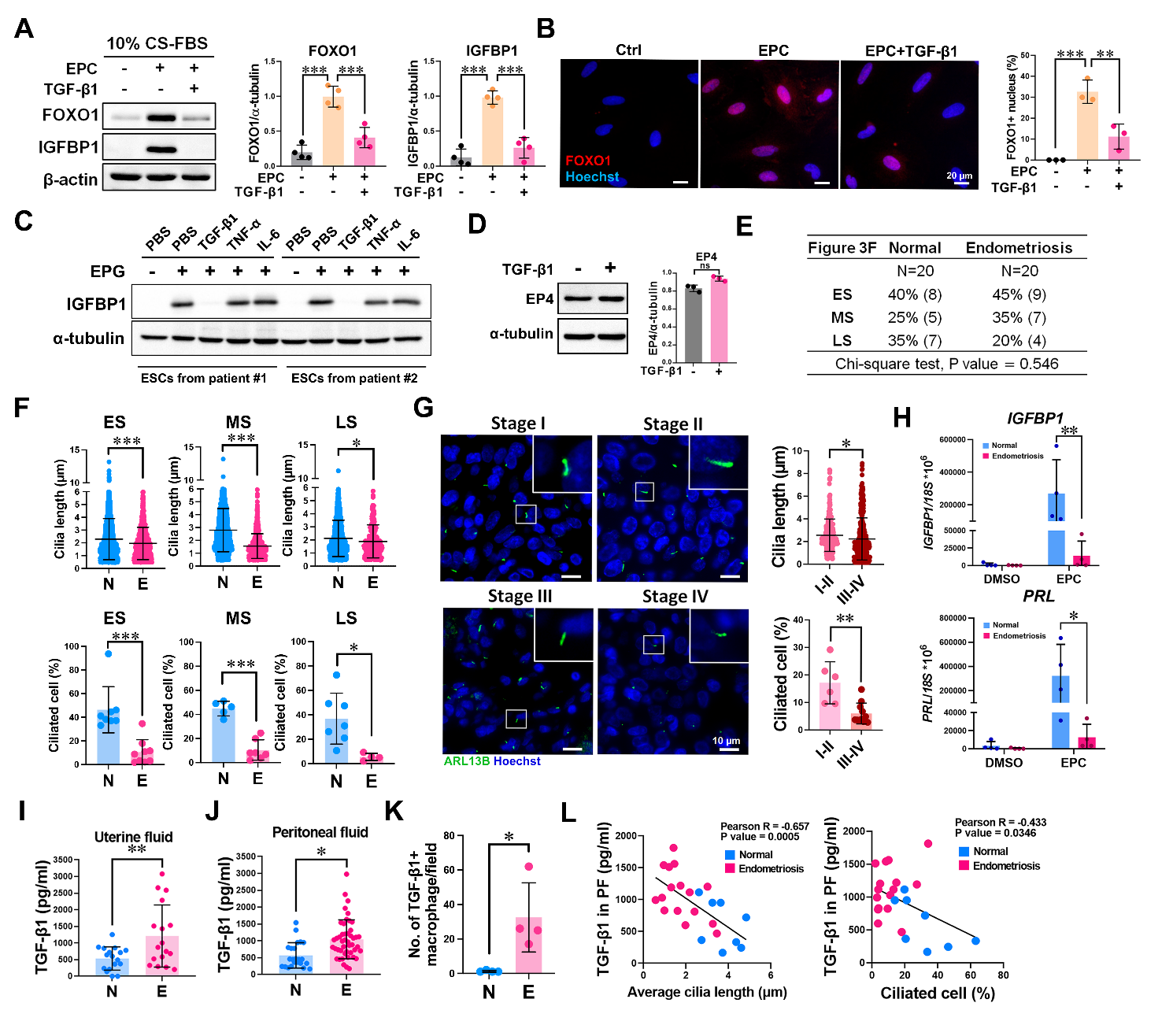


**Fig. S5. TGF-β1 negatively regulates ciliogenesis and decidualization in women with endometriosis. (A)** Representative Western blot images and quantified results of FOXO1 and IGFBP1 in ESCs treated with EPC medium combined with TGF-β1 (10 ng/ml) or vehicle under 10% charcoal-stripped (CS)-FBS conditions for 6 days (n=4 biological replicates). **(B)** Representative immunostaining images and quantitative results of FOXO1 (red) in the nuclei (blue) of ESCs (n=3 biological replicates). Scale bar = 20 μm. **(C)** Western blot images of IGFBP1 in ESCs treated with EPG medium combined with 10 ng/ml TGF-β1, TNF-α, or IL-6 for 4 days. The ESCs were derived from 2 women without endometriosis (normal). **(D)** Representative Western blot images and quantified results of EP4 in ESCs after TGF-β1 (10 ng/ml) treatment for 48 hours (n=3 biological replicates). **(E)** Distribution of early (ES), mid (MS), and late (LS) secretory phase samples in normal (N) and endometriosis (E) groups. Chi-square test shows no significant difference (P = 0.546). **(F)** Quantitative analysis of primary cilia length and percentage of ciliated cells in endometrial tissues from women with and without endometriosis. **(G)** Immunofluorescence images and analysis of primary cilia (anti-ARL13B, green) in endometria from endometriosis stages I-IV. Scale bar = 10 μm. Stages I and II: n=6; stages III and IV: n=12. **(****H)** The *IGFBP1* and *PRL* mRNA levels in ESCs isolated from the endometrium of women with or without endometriosis, treated with EPC medium or DMSO for 6 days (n = 4 biological replicates). **(I)** ELISA of TGF-β1 levels in uterine fluid from normal (N, n=17) and endometriosis (E, n=17) women. **(J)** ELISA of TGF-β1 in peritoneal fluid from normal (N, n=23) and endometriosis (E, n=45) women. **(K)** Quantitative analysis of TGF-β1-positive macrophages within the endometria of women with endometriosis (n=4) and without endometriosis (n=4). **(L)** Correlation of TGF-β1 concentration in peritoneal fluid with cilia length and the percentage of ciliated cells in endometria (normal: n=8; endometriosis: n=16). *p ≤ 0.05, **p ≤ 0.01, ***p ≤ 0.001 by one-way ANOVA followed by Tukey’s multiple comparison test (panels A and B), Student’s t-test, or Mann–Whitney U test (for two groups).

**
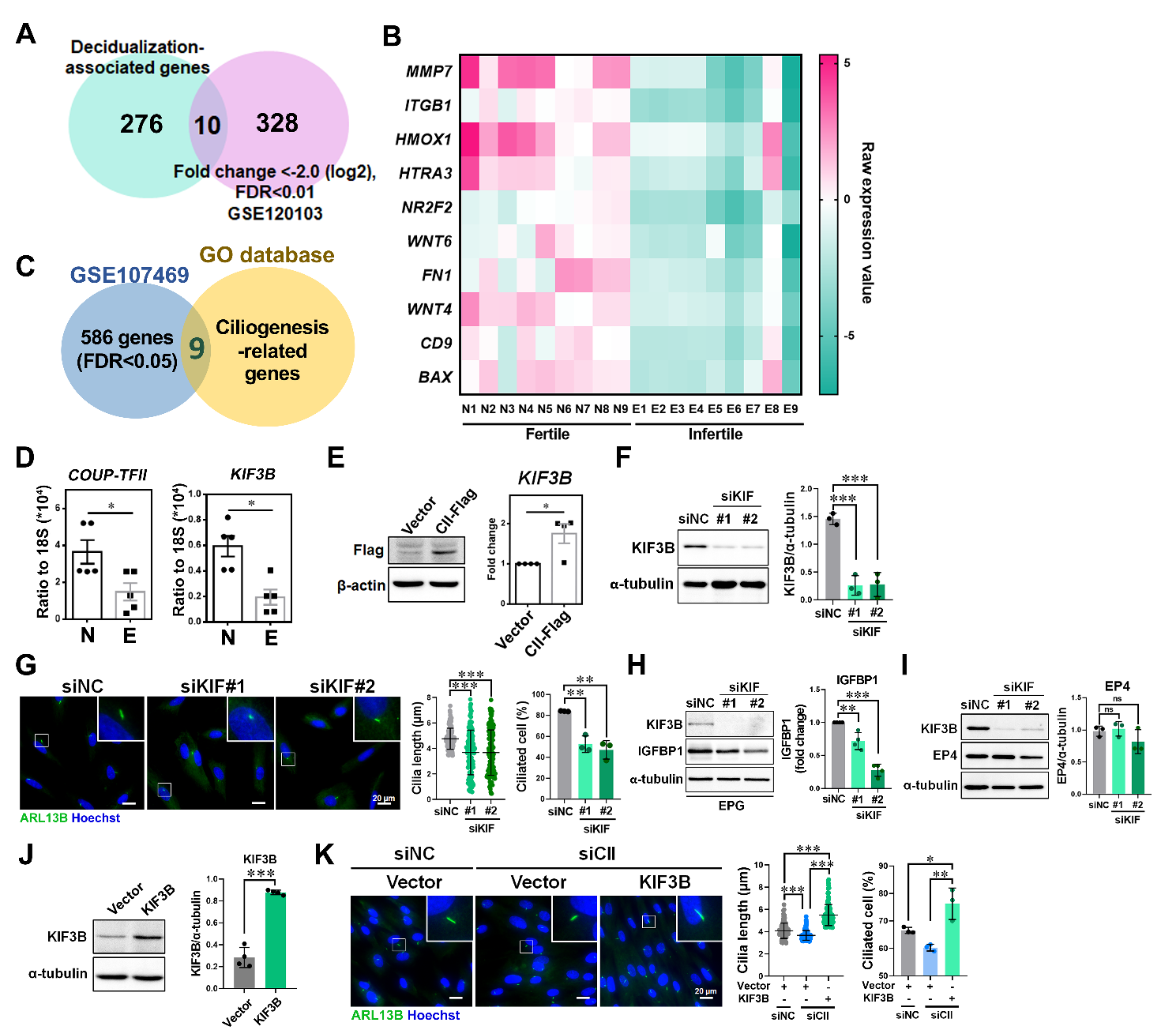
Fig. S6. COUP-TFII-regulated KIF3B expression contributes to primary cilia formation.** **(A)** Venn diagram showing the overlap between known decidualization-associated genes and differentially expressed genes (log2 FC < -2, FDR < 0.01) in the endometria of infertile women with endometriosis (GSE120103). **(B)** Heatmap of the top 10 differentially expressed decidualization-associated genes in endometria from fertile women and infertile women with endometriosis (GSE120103). **(C)** Venn diagram showing differentially expressed genes in *COUP-TFII* knockdown ESCs cross-referenced with ciliogenesis-related genes. **(D)** The mRNA levels of *COUP-TFII* and *KIF3B* in eutopic endometrial tissues from normal women (N, n=5) and women with endometriosis (E, n=5). **(E)** *COUP-TFII*-Flag (CII-Flag) plasmids or empty vector (pcDNA) were transfected into ESCs. Successful transfection was determined by immunoblotting for Flag, and the mRNA levels of *KIF3B* were analyzed (n=4 biological replicates). **(F)** Western blotting and quantification of KIF3B in *KIF3B*-knockdown (siKIF) and siNC ESCs (n=3 biological replicates). **(G)** Immunofluorescence and quantification of ciliogenesis in siKIF and siNC ESCs cultured in serum-free medium for 48 hours (n=3 biological replicates). Scale bar = 20 μm. **(H)** Western blotting and quantification of IGFBP1 levels in siKIF and siNC ESCs treated with an EPG medium for 4 days (n=4 biological replicates). **(I)** Western blotting and quantification of EP4 in siKIF and siNC ESCs (n=3 biological replicates). **(J)** Western blotting and quantification of KIF3B in *KIF3B* plasmids or empty vector-transfected ESCs.(n=4 biological replicates). **(K)** Representative immunofluorescence images and quantification of primary cilia (ARL13B, green) in ESCs transfected with control siRNA (siNC) or COUP-TFII siRNA (siCII), followed 24 h later by transfection with empty vector or a KIF3B expression plasmid. Cilia length and the percentage of ciliated cells were quantified (n = 3 biological replicates). Nuclei were stained with Hoechst (blue). Scale bar = 20 μm. Nuclei were stained with Hoechst (blue). Scale bar = 20 μm. *p ≤ 0.05, **p ≤ 0.01, ***p ≤ 0.001 by Mann–Whitney U test, Student’s t-test (for two groups), or one-way ANOVA followed by Tukey’s multiple comparison test (for three groups).


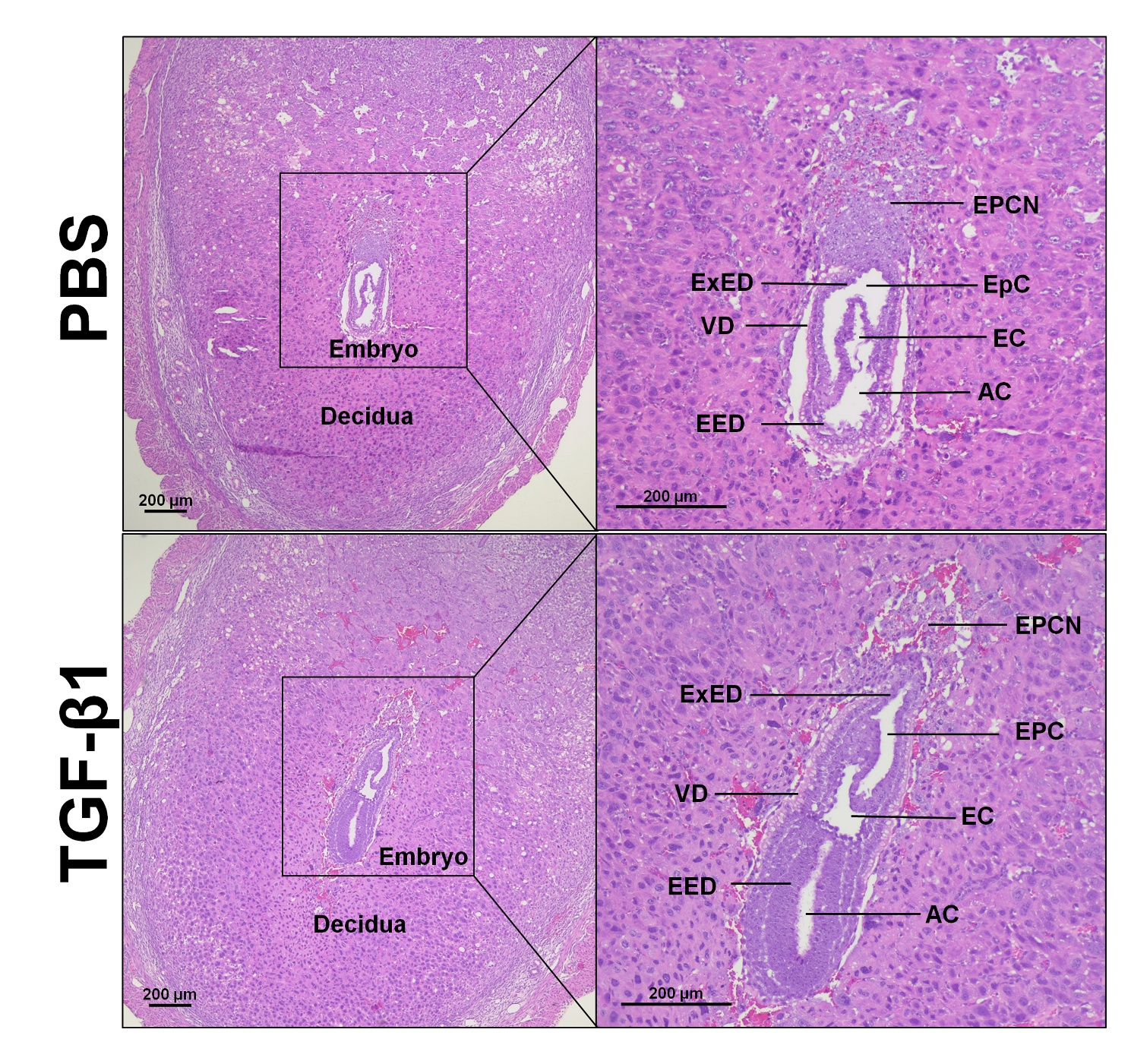


**Fig. S7. H&E staining of transverse sections of 7.5 dpc mouse embryos from mice administered with PBS (control) or TGF-β**1. The staining shows embryo implantation sites and decidua in the mouse uterus. Enlarged images of the embryos are shown on the right. Abbreviations: EPCN, ectoplacental cone; EPC, ectoplacental cavity; EC, exocoelomic cavity; AC, amniotic cavity; ExED, extraembryonic ectoderm; VD, visceral endoderm; EED, embryonic ectoderm. Scale bar = 200 μm.

**References:**

1. Inagaki N., Stern C., McBain J., Lopata A., Kornman L. and Wilkinson D. Analysis of intra-uterine cytokine concentration and matrix-metalloproteinase activity in women with recurrent failed embryo transfer. Hum. Reprod. 18(3):608-615, 2003.

2. Tsai S.J., Wu M.H., Lin C.C., Sun H.S. and Chen H.M. Regulation of steroidogenic acute regulatory protein expression and progesterone production in endometriotic stromal cells. J. Clin. Endocrinol. Metab. 86(12):5765-5773, 2001.

3. Wu M.H., Shoji Y., Wu M.C., Chuang P.C., Lin C.C., Huang M.F. and Tsai S.J. Suppression of matrix metalloproteinase-9 by prostaglandin E(2) in peritoneal macrophage is associated with severity of endometriosis. Am. J. Pathol. 167(4):1061-1069, 2005.
